# A Noncontiguous Code for RNA-Guided DNA Recognition Preceded CRISPR

**DOI:** 10.64898/2026.04.26.720920

**Published:** 2026-06-25

**Authors:** Peter H. Yoon, Kenneth Loi, Zeyuan Zhang, Trevor A. Docter, Santiago C. Lopez, Conner J. Langeberg, Muhammad Moezur-Rehman, Kamakshi Vohra, Zehan Zhou, Honglue Shi, Ron Boger, Peter Y. Wang, Benjamin A. Adler, Stephen G. Brohawn, Jennifer A. Doudna

**Affiliations:** 1 Department of Molecular and Cell Biology, University of California, Berkeley; Berkeley, CA, USA.; 2 Innovative Genomics Institute; University of California, Berkeley, CA, USA.; 3 Biophysics Graduate Group, University of California, Berkeley, Berkeley, CA, USA.; 4 California Institute for Quantitative Biosciences, University of California, Berkeley; Berkeley, CA, USA.; 5 Howard Hughes Medical Institute, University of California, Berkeley; Berkeley CA, USA.; 6 Department of Neuroscience, University of California, Berkeley; Berkeley, CA, USA; 7 Molecular Biophysics and Integrated Bioimaging Division, Lawrence Berkeley National Laboratory; Berkeley, CA, USA.; 8 Gladstone Institutes, University of California, San Francisco; San Francisco, CA, USA.; 9 Department of Chemistry, University of California, Berkeley; Berkeley, CA, USA.

## Abstract

CRISPR-Cas systems use RNA-guided proteins for adaptive immunity through a mechanism whose origin is unknown. Here we report the discovery of Viral Interference Programmable Repeat (VIPR) systems consisting of a Vipr protein more ancient than CRISPR-Cas and vrRNAs comprising alternating GGY/NN motifs. Unlike canonical guide RNAs that base pair with nucleic acid targets using an uninterrupted sequence, vrRNAs recognize double-stranded DNA through a noncontiguous code in which the variable NNs of each repeat collectively specify a target that itself contains a gapped recognition sequence. Analysis of natural vrRNA targets suggests VIPR acts against competing phages. We demonstrate programmable phage defense by redirecting the complex for transcriptional repression. These results suggest that the roots of adaptive immunity lie in ancient warfare between viruses, and reveal a new logic for programmable genetic control.

## Introduction

RNA-guided systems mediate diverse functions ranging from mobile genetic element propagation to adaptive immunity. These systems comprise proteins that use guide RNAs bearing sequence complementarity to nucleic acids, enabling programmable recognition of different substrates by the same protein. In all known RNA-guided proteins, target specificity is determined by contiguous base pairing between the guide and its target, and can be altered to direct the protein to a new substrate (1–4). In CRISPR-Cas systems, this versatility enables adaptive immunity in prokaryotes, and powers wide-ranging programmable biotechnologies that work across all domains of life (5, 6).

CRISPR-Cas systems comprise two classes based on their effector proteins (5, 7). Class 1 systems, the more abundant of the two, are thought to have evolved first (8). Their defining feature is Repeat-Associated Mysterious Proteins (RAMPs) that oligomerize on the CRISPR RNA (crRNA) to form the RNA-guided effector complex. RAMPs are the most conserved and ancient feature of CRISPR, proposed to date back to the last universal common ancestor (8, 9). Nevertheless, their origins remain unclear. We sought to identify ancestral RAMPs as a path to discovering the evolutionary origins of CRISPR.

## Results

### VIPR is a phage-encoded system with an ancestral RAMP protein

**Figure 1.**
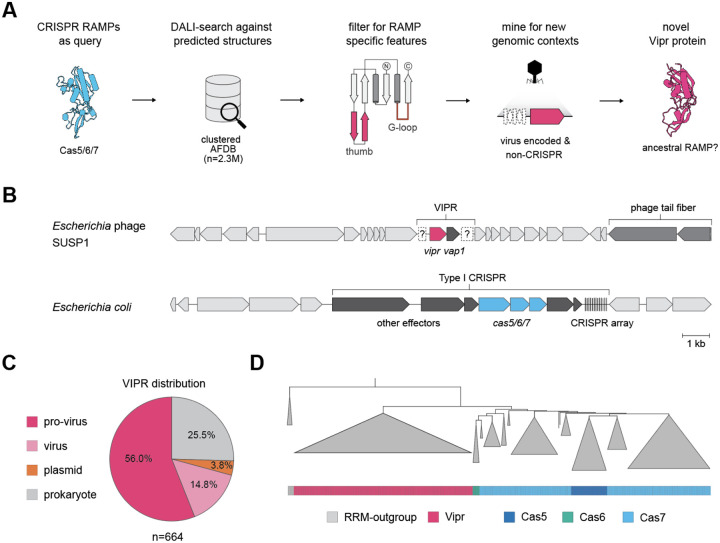
Vipr is a phage-encoded ancestor of CRISPR RAMPs. **(A)** Structure-guided discovery pipeline. **(B)** Comparison of a representative VIPR locus (*Escherichia* phage SUSP1) and a Type I CRISPR locus (*E. coli*). Question marks denote candidate noncoding RNA (ncRNA) regions. **(C)** Distribution of VIPR systems across viral and cellular genomes (n = 664). **(D)** Maximum-likelihood phylogenetic tree of Vipr and CRISPR RAMPs, rooted on an RRM outgroup.

RAMPs share little sequence similarity, rendering conventional homology search methods ineffective for identifying related proteins. Instead, we took advantage of shared RAMP structural features including the RNA Recognition Motif (RRM) core, the glycine-rich loop (G-loop), and the thumb domain that all mediate crRNA binding (fig. S1A) (8). These conserved features enabled structure similarity searches against the clustered AlphaFold database (Fig. 1A; table S1) (10–12). These searches revealed a large and previously unreported family of RAMP homologs encoded as part of a system we named Viral Interference Programmable Repeat (VIPR) (Fig. 1A; fig S1B, table S2).

Two features distinguish VIPRs from CRISPR systems. First, VIPR genomic loci are minimal, and typically include only two protein-coding genes, the *vipr* gene (the RAMP homolog) and a *vipr*-accessory protein (*vap*) (Fig. 1B). In contrast, CRISPR loci encode numerous *cas* genes (often multiple RAMPs), as well as CRISPR arrays (Fig. 1B). Notably, while VIPRs lack CRISPR arrays, the *vipr* gene is typically flanked by long intergenic sequences that could encode alternative noncoding RNAs (ncRNAs) distinct from crRNAs (Fig. 1B). Second, unlike CRISPR systems that occur primarily in cellular genomes, VIPRs are found primarily in bacteriophages and archaeal viruses (Fig. 1C). More specifically, most VIPRs are in proviral (56%) and viral (14.8%) genomes, although some are also found in prokaryotic chromosomes (25.5%) and plasmids (3.8%) (Fig. 1C; table S3). The distinct locus architecture and phage-encoded nature of VIPRs suggest their biological function and mechanism may be fundamentally different from those of CRISPRs.

Nevertheless, VIPRs and CRISPRs are related, and several lines of evidence suggest VIPRs are the more ancient form. Just as VIPR loci are minimal compared to CRISPR loci, Vipr proteins are primitive compared to CRISPR RAMPs, lacking the secondary RRM domain and other elaborations (fig. S1B). Moreover, Viprs show high sequence and taxonomic diversity (figs. S2, 3; table S3), and form an early branching clade separate from all CRISPR RAMPs (Cas5, Cas6, Cas7) (Fig. 1D; figs. S4, 5; data S1). Combining these observations, we propose that Vipr proteins represent an ancestral RAMP lineage predating the radiation of CRISPR-RAMPs. This conclusion implies that prokaryotes hijacked a viral protein to create the first CRISPR system.

### VIPRs encode a tandem repeat-containing RNA

**Figure 2.**
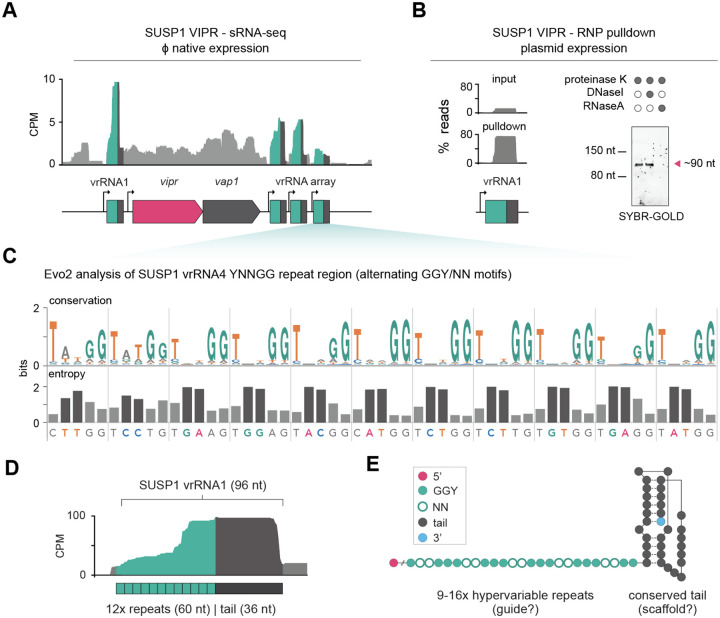
vrRNAs are tandem repeating ncRNAs. **(A)** Small RNA sequencing (sRNA-seq) of SUSP1 infected *E. coli* cells, 1 hour post-infection. Read coverage shown as counts per million (CPM). **(B)** sRNA-seq read distribution from input (top) and Vipr protein pull-down (bottom) after heterologous expression of SUSP1 VIPR in *E. coli*. Denaturing PAGE of copurified vrRNA across different enzyme treatment conditions (right). **(C)** Evo2-based conservation and entropy analysis of the SUSP1 vrRNA2 variable region. The primary sequence is shown below, with NN positions color-coded. (**D**) Zoom in of (A) on vrRNA1. Green boxes represent individual repeat units while the black rectangle indicates the 3’ tail. (**E**) Predicted secondary structure model of SUSP1 vrRNA.

Vipr proteins contain G-loop and thumb domains that in CRISPR RAMPs enable guide RNA recognition (fig. S1), which raises the possibility that Vipr proteins also utilize guide RNAs. To test this, we infected *E. coli* with the phage SUSP1 that natively encodes a VIPR system and performed small RNA (sRNA) sequencing (Fig. 2A). We detected four ~100-nucleotide transcripts encoded within the intergenic regions of the VIPR locus, hereafter called VIPR RNAs (vrRNAs) (Fig. 2A). One vrRNA was upstream, whereas three were in an array downstream of the protein-coding genes (Fig. 2A). *E. coli* plasmid-based expression of SUSP1 VIPR and the closely related *Pseudomonas fulva* prophage (hereafter *P. fulva*) VIPR also showed clear evidence of vrRNA expression (Fig. 2B; fig. S6). Moreover, vrRNAs of both systems co-purify with their respective Vipr proteins, suggesting that the two form a stable ribonucleoprotein (RNP) complex (Fig. 2B; fig. S6).

Analysis of vrRNA sequences using the genomic language model Evo2 (13) revealed that vrRNAs comprise 9–16 YNNGG tandem repeats in which conserved a GGY motif (Y = pyrimidine; N = any base) alternates with hypervariable NN dinucleotides (Fig. 2C; fig. S7). Evo2 yielded conservation scores that were high for the GGY segments, but low for the NN segments, with correspondingly high-entropy scores for the latter (Fig. 2C; fig. S7). Consistent with this, manual inspection of 137 unique vrRNA sequences from SUSP1 and *P. fulva* VIPR clades confirmed that all vrRNAs maintained the conserved GGY motifs while the NN bases were hypervariable (fig. S8).

The tandem repeat tract of vrRNAs precedes an invariable 3' extension hereafter referred to as the tail (Fig. 2D). RNA-folding analysis suggests that the tail adopts a conserved secondary structure that includes a pseudoknot in the SUSP1 VIPR clade (Fig. 2E, fig. S9). This two-part organization where a variable region is capped by a structurally conserved region parallels crRNAs, in which the programmable spacer is capped by the repeat handle.

### vrRNAs use a tandem repeat code to recognize DNA

We wondered whether the similar architecture of vrRNAs and crRNAs reflects a role of vrRNAs in nucleic acid targeting. Considering how alternating GGY/NN motifs could confer target specificity, we hypothesized that the conserved GGY positions do not participate in base pairing, while the variable NN positions specify the nucleic acid target. This was inspired by Class 1 CRISPR, where RAMP subunit thumb domains disrupt basepairing at every sixth position of the crRNA-target duplex (14). VIPR subunit thumb domains could similarly preclude basepairing at every GGY position along the vrRNA-target duplex and position the hypervariable NN nucleotides spatially for target recognition.

**Figure 3.**
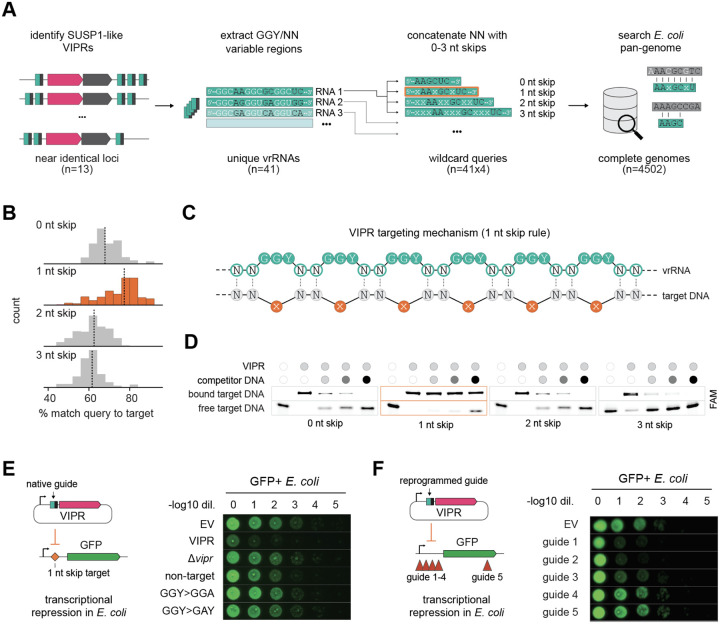
vrRNAs recognize DNA using a non-contiguous base pairing code. (**A**) Computational pipeline to identify vrRNA targeting rules. Four possible non-contiguous recognition patterns where vrRNA’s GGY/NN motifs recognizes NN (0 nt skip), xNN (1 nt skip), xxNN (2 nt skip), or xxxNN (3 nt skip) were tested. (**B**) NN match frequency distributions for each skip rule. Each alignment to the *E. coli* pan-genome is scored by the percentage of matching vrRNA NN positions (rightward shift indicates higher match quality). **(C)** VIPR targeting mechanism. Every NN position of vrRNA base-pairs with the target using the 1 nt skip rule. The orange base corresponds to the “skip base”. **(D)** *In vitro* competitive binding assay with SUSP1 VIPR (Vipr-vrRNA ribonucleoprotein) using different skip rule substrates (fluorescein-labeled; FAM) and salmon sperm competitor DNA. The competitor to substrate ratio is 0, 1, 2, and 10 fold excess by weight. **(E)** VIPR mediated green fluorescent protein (GFP) repression assay. Empt vector (EV) was used as negative control. **(F)** GFP repression assay results with reprogrammed vrRNAs.

We tested this model of non-contiguous base pairing by searching the *E. coli* pangenome using inferred targets of SUSP1 vrRNAs (Fig. 3A). For each vrRNA, we concatenated the NN dinucleotides with 0–3 intervening wildcard bases (i.e., NN, xNN, xxNN, or xxxNN) to model targets with different “skip base” lengths (Fig. 3A). We hypothesized that skip bases range from zero if NN dinucleotides are read directly without gaps to three if non-base-paired positions mirror the full GGY trinucleotide. Targets modeled with a single skip base (xNN; the “1 nt skip rule”) yielded substantially more high-quality matches than any alternative (Fig. 3B, C; data S2). Among the top matches, most inferred targets perfectly matched protein coding sequences with skip bases overwhelmingly aligning to the third (wobble) position of codons (fig. S10). These inferred target sites could be found on both strands (fig. S10), consistent with DNA targeting by VIPR systems.

Both *in vitro* biochemical and *in vivo* functional assays confirmed the validity of the 1 nt skip rule. We reconstituted SUSP1 VIPR RNP *in vitro* and tested binding to double-stranded DNA targets designed with 0–3 nt skip lengths (Fig. 3D; fig. S11). The RNP bound all four substrates in the absence of a competitor, but in the presence of excess competitor DNA, only the 1 nt skip substrate remained bound. We corroborated this result *in vivo* by placing target sites following 0–3 nt skips upstream of a green fluorescent protein (GFP) reporter (Fig. 3E), reasoning that specific binding would repress transcription as in CRISPRi (15). Only the 1 nt skip target sequence caused GFP repression (Fig. 3E; fig. S12A), and deletion of *vipr* or perturbation of vrRNA GGY motifs or NN basepairing abolished silencing (Fig. 3E; fig. S12B). Unlike dCas9-based CRISPRi, targeting either coding or template strands silenced GFP expression (Fig. 3E; fig. S12B) (15).

VIPR is a programmable system in which vrRNAs dictate target specificity. Testing different native SUSP1 vrRNAs confirmed that each RNA silenced GFP expression when its predicted target was placed upstream of the GFP reporter (fig. S12C). Moreover, specificity is entirely reprogrammable by altering only the NN dinucleotide regions. Three of four vrRNAs reprogrammed to promoter proximal positions effectively repressed GFP (Fig. 3F). Target recognition did not require sequence constraints beyond the 1 nt skip rule, lacking a PAM-like motif found in CRISPR-Cas enzymes as predicted by our bioinformatic analysis (fig. S13). Together, these results establish that VIPR recognizes DNA through non-contiguous base pairing, with target specificity encoded entirely by the NN dinucleotides of the alternating GGY/NN motifs.

### VIPRs repress competing mobile genetic elements

**Figure 4.**
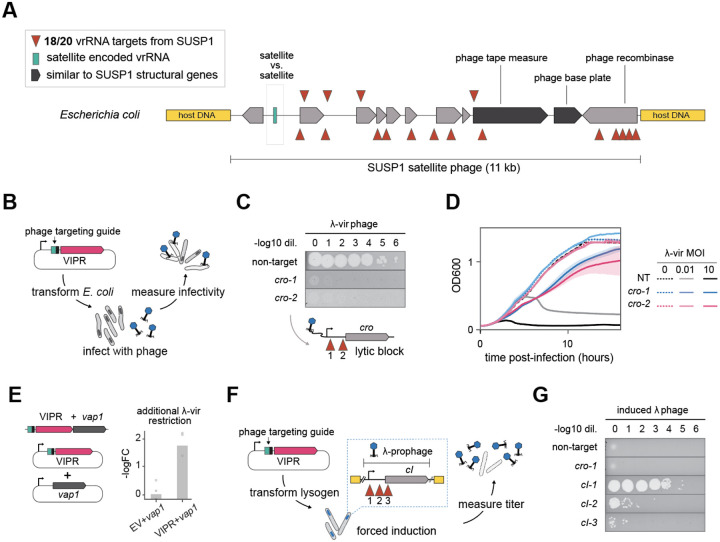
VIPRs control phage behavior. **(A)** SUSP1 vrRNA targets mapped onto the SUSP1 satellite phage locus in the *E. coli* genome. **(B)** Schematic of phage defense assay. **(C)** Plaque assay results with indicated cro-targeting guides. Below, guide positions in *cro*. **(D)** Liquid culture defense assay at indicated MOIs. Shaded regions, s.d. (n=3). (**E**) Relative enhancement of phage-restriction conferred by *vap1* co-expression relative to empty vector (EV), in the absence or presence of VIPR. **(F)** Schematic of prophage induction assay. **(G)** Plaque assay results from (F) with indicated guides.

Decoding the tandem repeat specificity rule allowed us to predict natural vrRNA targets, revealing VIPR to be an agent of inter-phage warfare. In the SUSP1 VIPR clade, 18 of 20 vrRNA targets converge on a satellite phage that likely parasitizes SUSP1 (Fig. 4A; data S3), as inferred by shared homology between their structural genes (fig. S14). Some of these satellites encode their own vrRNAs directed at other closely related satellites, revealing layers of conflict among competing elements (Fig. 4A; data S3). In the *P. fulva* VIPR clade, 48 of 66 vrRNA targets map to resident *Pseudomonas* prophages (data S3). 36 vrRNAs concentrate on a single prophage locus, primarily targeting transcriptional regulators of the lysogeny-lytic switch (fig. S15; data S3). Notably, some vrRNAs target other VIPR systems, suggesting they are deployed defensively as well (fig. S15; data S3). VIPR thus operates as both sword and shield in inter-phage conflict.

We tested two scenarios to examine whether VIPR can control the behavior of phages. First, we tested VIPR’s ability to defend against an invading phage. We challenged *E. coli* expressing a VIPR system from a plasmid with vrRNAs targeting the essential lytic regulator *cro* in an obligately lytic λ variant (Fig. 4B). *cro*-targeting guides reduced λ phage plaquing efficiency by five orders of magnitude and conferred immunity in liquid culture, while a non-targeting guide conferred no protection (Fig. 4C, D). Notably, co-expression of *vap1* from the SUSP1 VIPR operon further enhanced protection (Fig. 2A, 4E). Second, we tested VIPR’s ability to force a resident prophage into lysis by targeting the lysogenic regulator *cI* in an *E. coli* λ-lysogen (Fig. 4F). *cI*-targeting vrRNAs triggered prophage induction, while *cro*-targeting and non-targeting vrRNAs produced no observable induction (Fig. 4G). Collectively, these results demonstrate that VIPR is a flexible system for control over inter-phage conflict.

### Diverse VIPR systems link inter-phage conflict to adaptive immunity

**Figure 5.**
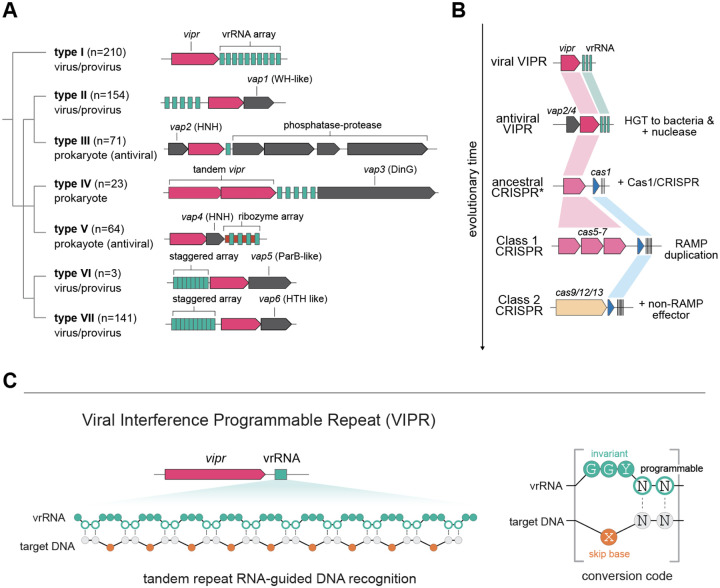
Diverse VIPR systems suggest a new model for CRISPR evolution. **(A)** Classification of 667 representative VIPR systems into seven types. Simplified Vipr protein phylogeny annotated with type, abundance, and taxonomic distribution (left). Representative locus diagrams for the type (right). Green squares, vrRNAs; magenta, *vipr* gene; gray, *vap* gene*s*. (**B**) Diagram of proposed model for VIPR evolution to CRISPR via horizontal gene transfer (HGT) from virus to prokaryotic host. (*) denotes hypothetical locus. (**C**) Summary of VIPR RNA-guided DNA recognition mechanism following the 1 nt skip rule.

VIPRs are diverse systems built around the tandem repeat recognition code. VIPRs comprise seven distinct types defined by Vipr protein phylogeny, vrRNA architecture and accessory gene associations (Fig. 5A; fig. S16; data S4). Although all seven types use vrRNAs comprising alternating GGY/NN motifs, or a related consensus (fig. S17), array architectures are variable (Fig. 5A). Beyond the array architectures in type II VIPR systems characterized here, there exist staggered arrays with overlapping vrRNAs (fig. S18), hammerhead ribozyme-interspersed arrays (fig. S19), as well as systems that only encode one vrRNA. The latter two were confirmed experimentally using sRNA-sequencing (fig. S20). Likewise, although all six *vap* genes (*vap1-6*) appear to be involved in DNA interaction, their predicted functions are variable (Fig. 5A). In some systems, as in the case of type II, VI, and VII, *vap* genes encode predicted DNA binding proteins, whereas types III-V encode predicted enzymes such as DNA nucleases or helicases (fig. S21).

Some VIPR systems appear to have been repurposed from inter-viral warfare to host-virus warfare. For instance, types III and V are enriched in defense islands and their vrRNAs target viral sequences, suggesting that they perform host immune functions (fig. S22; data S3). Notably, these antiviral VIPRs are the only types that encode predicted nucleases, suggesting their primary mode of activity is target destruction rather than repression. These observations imply that the ancestral viral targeting function of VIPR persisted following horizontal transfer to prokaryotic genomes, and prokaryotes repurposed these from inter-phage competition to host anti-phage immunity by gaining new *vap* genes (Fig. 5B). The direct ancestors of modern CRISPR systems may have followed a similar trajectory to give rise to adaptive immunity.

## Discussion

VIPR systems constitute a previously unknown mechanism of RNA-guided DNA recognition for genetic control that appears to pre-date the emergence of CRISPR pathways. Unlike all other known types of guide RNAs in which contiguous segments determine target specificity, vrRNAs instead encode targeting information in the hypervariable positions of tandem pentanucleotide repeats. Vipr proteins use this code, in the form of a gapped array of NN dinucleotides within the 9–16 YNNGG vrRNA tandem repeats, for noncontiguous DNA target recognition with a periodic one-nucleotide skip. This encode-decode logic, in which a repeating RNA scaffold stores sequence specificity that is interpreted by its protein partner, represents a distinct solution to programmable nucleic acid recognition.

The finding of at least seven different types of VIPR systems within bacteriophage and prokaryotic genomes, many of which target other phages, implies an ancient and widespread network for programmable virus genetic control. In at least some cases, VIPRs are anti-phage systems used for control of phage propagation by targeted transcriptional silencing. In principle, phage can develop resistance to direct sequence targeting by acquiring mutations in synonymous codons with variation at the third codon nucleotide, the wobble position. Notably, in VIPR systems, the skipped nucleotide in the target DNA generally corresponds to the codon wobble position. This skipping logic would allow VIPR systems to overcome possible viral resistance by synonymous codon swaps.

Based on these observations, we propose that bacteria co-opted ancestral VIPR-like systems used in inter-phage warfare for host defense (Fig. 5B; fig. S22). Coupling of such defense-associated VIPRs with a Cas1-like transposase would have enabled heritable immune memory, giving rise to the first CRISPR system (16). Subsequent duplication and diversification of the ancestral RAMP could have then produced the specialized Class 1 CRISPR RAMPs (8). Finally, replacement of RAMP effectors with non-RAMP effectors might then have led to Class 2 CRISPR systems (8). In this model, VIPRs are more ancient than CRISPRs and viral conflict seeded the evolution of CRISPR-Cas adaptive immunity.

VIPR systems require only a single small protein (~20 kD) and a single guide RNA (<100 nucleotides). They recognize target sequences located on either DNA strand of a gene without a PAM constraint, and are readily reprogrammable. These properties make VIPRs among the most minimal and potentially versatile platforms for RNA-guided DNA recognition yet described. In addition to transcriptional regulation, synthetic Vipr fusions could enable applications including genome editing, DNA locus imaging and epigenetic modification. More broadly, the discovery of a new code for RNA-mediated sequence recognition expands the known design space for programmable genetic control and suggests that further modes of RNA-guided recognition remain to be found.

## Supplementary Material

1

Materials and Methods

Supplementary Text

Figs. S1 to S22

Tables S1 to S3

References (1–24)

Data S1 to S4

## Data Availability

All data, code, and materials used in the analysis will be made publicly available online.
