# Supplementary material for: A Noncontiguous Code for RNA-Guided DNA Recognition Preceded CRISPR
Source: bioRxiv. 2026 Jun 25:2026.04.26.720920. Originally published 2026 Apr 27. Preprint. [Version 2] doi: 10.64898/2026.04.26.720920 (PMC13142463; doi:10.64898/2026.04.26.720920)
Supplement: 1 [file NIHPP2026.04.26.720920V2-supplement-1.pdf]

bioRxiv preprint doi: https://doi.org/10.64898/2026.04.26.720920; this version posted June 25, 2026. The copyright holder for this preprint (which was not certified by peer review) is the author/funder, who has granted bioRxiv a license to display the preprint in perpetuity. It is made available under a CC-BY 4.0 International license.

Supplementary Materials for

A Noncontiguous Code for RNA-Guided DNA Recognition Preceded CRISPR

Authors: Peter H. Yoon[1,2†], Kenneth Loi[1,2†], Zeyuan Zhang[2,3], Trevor A. Docter[1], Santiago C. Lopez[1], Conner J. Langeberg[1,4], Muhammad Moez ur-Rehman[1,2], Kamakshi Vohra[1,4‡], Zehan Zhou[1,2], Honglue Shi[1,4], Ron Boger[2,3], Peter Y. Wang[2,5], Benjamin A. Adler[2], Stephen G. Brohawn[1,4,6] and Jennifer A. Doudna[1-5,7-9*]

*Corresponding author. Email: doudna@berkeley.edu

The PDF file includes:

Materials and Methods
Figs. S1 to S22

bioRxiv preprint doi: https://doi.org/10.64898/2026.04.26.720920; this version posted June 25, 2026. The copyright holder for this preprint (which was not certified by peer review) is the author/funder, who has granted bioRxiv a license to display the preprint in perpetuity. It is made available under a CC-BY 4.0 International license.

Materials and Methods

VIPR system discovery and identification pipeline

VIPR systems were identified following the structure-search pipeline previously reported (*12*). CRISPR RAMPs including Cas5 (PDB ID: 6IFN, chain B), Cas6 (PDB ID: 6FJW, chain A), and Cas7 (PDB ID: 6IFN, chain E) were used as queries for DALI searches (*10*) against the clustered AlphaFold Database (clustered AFDB) (*11*). Hits were first filtered for a Z-score of at least 6, and then inspected for the thumb domain and glycine rich loop (G-loop), the hallmark features of RAMPs. The genomic loci of these RAMP homologs were examined using fast.genomics to identify RAMPs found in non-CRISPR contexts (*16*). This revealed that five hits (UniProt IDs: X1U2U4; A0A0F9A1L0; A0A661D0E2; A0A1F4YLN4; A0A2D6XG06) exist as either stand-alone genes or in simple two-gene operons not associated with CRISPR repeats, corresponding to VIPR systems reported in this study.

X1U2U4 was then used as a query for PSI-BLAST against the NCBI NR database through the MPI Bioinformatics Toolkit (8 iterations, maximum 10,000 target hits). This yielded 2,664 unique Vipr protein sequences. For each PSI-BLAST hit, the genomic sequence corresponding to a 20 kb window centered on the hit was retrieved from NCBI, discarding contigs shorter than 20 kb. Resulting contigs were deduplicated using MMseqs2 (18) at the nucleotide level (min-seq-id 0.5, -c 0.5). This yielded 664 representative loci and 667 distinct VIPR systems and 670 Vipr proteins referenced throughout the paper. Genome types of these representatives were classified using geNomad (19) in end-to-end mode. Contigs called plasmids by geNomad were classified as plasmids. For contigs called viruses by geNomad, NCBI taxonomy was used to distinguish free viruses (NCBI assignment to Viruses) from proviruses (NCBI assignment to a cellular lineage). All remaining loci were classified as cellular.

bioRxiv preprint doi: https://doi.org/10.64898/2026.04.26.720920; this version posted June 25, 2026. The copyright holder for this preprint (which was not certified by peer review) is the author/funder, who has granted bioRxiv a license to display the preprint in perpetuity. It is made available under a CC-BY 4.0 International license.

RAMP and VIPR phylogenetic analysis

CRISPR RAMP DALI hits were pooled with a representative subset of Vipr proteins mined from NCBI. The resulting dataset was aligned using the DALI/T-COFFEE structure based multiple-sequence alignment pipeline previously described (12). Incomplete proteins that did not span the full length of the RAMP fold as inferred by the DALI/T-COFFEE alignment were dropped from the dataset. The alignment was then manually trimmed to only include RAMP core structural features corresponding to helices α1–α2, strands β1–β4, the thumb domain, and the glycine-rich loop.

Phylogenetic trees were inferred using IQ-TREE v3.0.1 (-bb 1000 -alrt 1000) (*17*) . Three substitution models of increasing levels of complexity were tested (MFP/BLOSUM62, LG+F+R6, and LG+C60+F+R6). The resulting trees were re-rooted using a non-RAMP member of the RRM superfamily as an outgroup. Tree robustness was further tested by subsetting the dataset using MMseqs2 based deduplication on the trimmed alignment (min-seq-id 0.8 and 0.5, both with -c 0.8), as well as further trimming the alignment using trimAl (*18*) (-gt 0.5 and -gt 0.3). Tree leaves were annotated using a curated set of CRISPR-RAMP HMM profiles (*19*) using hmmsearch (*20*), with each sequence annotated based on the highest-scoring profile hit.

VIPR type classification

The 667 representative VIPR systems identified from NCBI were used for type classification. Vipr protein phylogeny was first constructed to provide the primary framework for classification. HHalign was used to align the representative Vipr proteins to a seed alignment generated using the DALI/T-COFFEE method, and phylogeny was inferred with IQ-TREE under the LG+F+R6 model. Contigs encoding the 667 VIPR systems were sorted based on their phylogeny. Protein-coding sequences across all loci were clustered as previously described (*21*),

bioRxiv preprint doi: https://doi.org/10.64898/2026.04.26.720920; this version posted June 25, 2026. The copyright holder for this preprint (which was not certified by peer review) is the author/funder, who has granted bioRxiv a license to display the preprint in perpetuity. It is made available under a CC-BY 4.0 International license.

and cluster assignments were mapped onto GenBank records as gene annotations. Annotated loci were visually inspected in Geneious to identify gene synteny patterns and *vap* genes for each VIPR type. Candidate vrRNAs were identified by searching for YNNGG tandem repeats with at least four repeat units (low repeat threshold to account for the frequently observed internal degeneracy of vrRNAs). For loci lacking recognizable YNNGG repeats, candidate vrRNAs were identified based on Evo2 scoring patterns. This revealed alternative vrRNAs comprising NNNGG, YNNNG, and NNNNG repeats. vrRNA architecture was typified for a given type when the pattern was observed across multiple independent members of the clade.

Bacterial strains and culturing

NEB 10-beta *E. coli* (New England Biolabs), 10-beta *E. coli* Mix & Go! Competent Cells (Zymo), and Mach1 *E. coli* (Invitrogen) were used for plasmid cloning and all experiments in this study. *E. coli* MG1655 was used as an indicator strain to quantify the efficiency of λ prophage induction from a lysogenised MG1655 strain, as described below. *E. coli* strains were grown in LB broth at 37°C, 250 RPM or on 1.5% agar plates, with appropriate inducers and antibiotics. Inducers and antibiotics were used at the following working concentrations: 0.002% (GFP repression assays) or 0.2% (ribonucleoprotein or RNP purification) L-arabinose, 100 (phage assays) or 500 (RNP purification) nM crystal violet (Sigma), 35 µg/mL kanamycin (Sigma) and 100 µg/mL ampicillin or carbenicillin (Sigma).

Cloning and plasmid construction

All plasmids were assembled by Gibson assembly, Golden Gate assembly, or KLD/primer-extension cloning. PCR products were generated with KAPA HiFi (Roche) or PrimeStar GXL (Takara) PCR mixes using IDT primers. Final constructs were sequence-verified

bioRxiv preprint doi: https://doi.org/10.64898/2026.04.26.720920; this version posted June 25, 2026. The copyright holder for this preprint (which was not certified by peer review) is the author/funder, who has granted bioRxiv a license to display the preprint in perpetuity. It is made available under a CC-BY 4.0 International license.

by Plasmidsaurus or Quintara Biosciences. Sequences of constructs will be provided in the final publication. Two expression systems were used throughout this study. The pJEX-SC101 vector includes the crystal violet inducible pJEX promoter. The pBAD-ColE1 vector includes the arabinose-inducible pBAD promoter. The SUSP1 VIPR locus comprising vrRNA1 and *vipr* CDS was PCR amplified from the SUSP1φ genome. This sequence is hereafter called “VIPR” when referencing effector plasmids.

For experiments involving RNP purification, VIPR was cloned into the pJEX-SC101 vector with a Twin-Strep tag or a 6xHis tag on the C-terminus of the Vipr protein. For the equivalent experiments for *Pseudomonas fulva* (*P. fulva)* VIPR, the entire locus spanning vrRNA1-3, *vipr*, and the *vap1* gene was cloned into the pBAD-ColE1 vector with a C-terminal Twin-Strep tag on Vipr. For RNP-based small RNA-sequencing experiments, the Twin-Strep tag constructs were used. For RNP used in *in vitro* binding assays, the 6xHis-tagged constructs were used.

For the GFP repression assays, VIPR was cloned into the pBAD-ColE1 vector. This parent plasmid was used to generate mutant variants designed to test sequence requirements of the VIPR system. The reprogrammed GFP-targeting vrRNA variants were generated from this parent by altering only the NN regions of vrRNA1. Plasmids referred to as “native guide 2” and “native guide 3” were cloned by replacing vrRNA1 with either the downstream vrRNA2 or 3.

The GFP target plasmids express sfGFP from the constitutive J23119 promoter on a SC101 vector. To test native guides, the cognate vrRNA target sites were inserted into the 5′ UTR between the promoter and the sfGFP CDS. To test the skip rule, predicted vrRNA1 target sites with gap lengths of 0-3 nt were inserted at the same location. In all cases, "G" was used as the skip bases. For the reprogrammed guide assays, the parent GFP target plasmid without any inserted target site in the 5′ UTR was used.

bioRxiv preprint doi: https://doi.org/10.64898/2026.04.26.720920; this version posted June 25, 2026. The copyright holder for this preprint (which was not certified by peer review) is the author/funder, who has granted bioRxiv a license to display the preprint in perpetuity. It is made available under a CC-BY 4.0 International license.

For plaque and liquid infection assays against λvir-cro, VIPR was cloned into the pJEX-SC101 vector, and vrRNA1 was reprogrammed to target the *cro* regulator. To assay combined activity of VIPR with the *vap1* gene, pBAD VIPR was co-transformed with the *vap1* gene cloned into a pJEX-SC101 vector. For VIPR-targeted prophage induction in *E. coli* MG1655::λbor::kan, the pBAD VIPR with vrRNA1 reprogrammed to target the *cI* regulator was used.

Small RNA sequencing

RNA was extracted using the hot formamide method. For total RNA, *E. coli* pellets were resuspended in 18 mM EDTA and 95% formamide at 65ºC for 5 minutes. Lysed cell solution was then clarified via centrifugation at 11 kg for 1 minute, and total RNA was purified using the RNA Clean & Concentrator-5 kit (Zymo) following manufacturer protocol. For RNAs co-purified with Vipr protein, purified VIPR RNP was used instead of *E. coli* pellets as input, but otherwise subject to the same purification procedure. For rRNA depletion, approximately 200 ng of RNA underwent treatment using the NEBNext rRNA kit (New England Biolabs). RNA was purified using SPRI-select following a 1:2:2 volumetric ratio of RNA, SPRI beads, and isopropanol. RNA ends were repaired in T4 PNK buffer (New England Biolabs) by sequential treatment with Quick CIP (New England Biolabs) at 37ºC for 10 minutes to remove 5′ and 3′ phosphates, followed by heat inactivation at 80ºC for 2 minutes, then T4 PNK (New England Biolabs) at 37ºC for 30 minutes to resolve 2′,3′-cyclic phosphates. ATP was spiked into the mixture to a final concentration of 10 mM to phosphorylate 5′ ends, and further incubated at 37ºC for 30 minutes. RNA was again purified using SPRI-select at 1:2:2 ratio, and used as input for Collibri small-RNA-seq library preparation following manufacturer protocol. Adapted ligated cDNA was separated on 4% E-Gel EX Agarose Gels, and size-selected for a 150–400 nt range via gel

bioRxiv preprint doi: https://doi.org/10.64898/2026.04.26.720920; this version posted June 25, 2026. The copyright holder for this preprint (which was not certified by peer review) is the author/funder, who has granted bioRxiv a license to display the preprint in perpetuity. It is made available under a CC-BY 4.0 International license.

extraction. Sequencing was performed on Illumina platforms with 150 bp paired-end reads. Reads were imported to Geneious, merged using BBMerge (*22*), and mapped to their respective reference loci using Geneious Mapper.

Sequence Logo Generation

Evo2 sequence scores were obtained by querying the hosted Evo2 model through NVIDIA's authenticated NIM API and extracting forward-pass logits for each input DNA sequence. At each position, logits were converted to probabilities with a full-vocabulary softmax, and the probabilities for *A*, *C*, *G*, and *T* were extracted.

Shannon entropy was calculated as:

$$H_i = -\sum_b p_{i,b} \log_2 p_{i,b}$$

Information content was calculated as:

$$I_i = 2 - H_i$$

Per-base logo heights were calculated as:

$$h_{i,b} = p_{i,b} I_i$$

Because Evo2 outputs next-token predictions, score and logo tracks were shifted by +1 nucleotide to align predictions with the scored nucleotide.

MSA logo plots were calculated analogously, but base probabilities were estimated empirically from aligned sequence columns rather than model logits. At each alignment position, counts of *A*, *C*, *G*, and *T* were tabulated across sequences. Smoothed base frequencies were computed as

$$p_{i,b} = \frac{n_{i,b} + 0.25}{N_i + 1}$$

bioRxiv preprint doi: https://doi.org/10.64898/2026.04.26.720920; this version posted June 25, 2026. The copyright holder for this preprint (which was not certified by peer review) is the author/funder, who has granted bioRxiv a license to display the preprint in perpetuity. It is made available under a CC-BY 4.0 International license.

where $n_{i,b}$ is the count of base b and N_i is the total number of non-gap canonical bases at that position. Entropy, information content, and logo heights were then computed using the same formulas described above. For alignment heatmaps, each column corresponds to an alignment position and rows *A*, *C*, *G*, and *T* display the smoothed per-position nucleotide frequencies used for the logo calculations, with shading scaled from 0 to 1.

vrRNA secondary structure analysis

The SUSP1 Vipr protein was used as a BLASTp query against NCBI NR to identify near-identical VIPR systems (>90% protein identity), resulting in 13 loci that define the SUSP1 VIPR clade. From these loci, 41 unique vrRNAs were manually identified. The conserved 3' tail region (~40 nt downstream of the last YNNGG repeat) was extracted from five diverse SUSP1 VIPR clade vrRNA sequences and aligned without gaps. Individual sequences and subsequences were folded using RNAfold (*23*) to identify recurrent predicted structural motifs. A candidate secondary structure was then evaluated by manually introducing gaps into the alignment. Compensatory mutations at predicted base-paired positions were identified by visual inspection to infer the final structure model.

Informatic prediction of VIPR targeting rule

Under a model where the vrRNA YNNGG repeat region (e.g., GGY/NN alternating motifs) encodes target specificity solely through the NN dinucleotides, two possible target architectures come to mind. First is a compact target where the NN dinucleotides are directly concatenated. vrRNA-target duplex then would form by extrusion of the GGY positions (guide: 5′-GGYNN-GGYNN-GGYNN-3′; target: 3′-NN-NN-NN-5′). Second is a symmetric target where successive NN dinucleotides are separated by three "skipped" bases ("x") that do not basepair

bioRxiv preprint doi: https://doi.org/10.64898/2026.04.26.720920; this version posted June 25, 2026. The copyright holder for this preprint (which was not certified by peer review) is the author/funder, who has granted bioRxiv a license to display the preprint in perpetuity. It is made available under a CC-BY 4.0 International license.

with the guide GGY positions. vrRNA-target duplex then would form by mismatches at the GGY positions (guide: 5′-GGYNN-GGYNN-GGYNN-3′; target: 3′-xxxNN-xxxNN-xxxNN-5′). Between these extremes, the number of skipped bases could be one (3'-xNN-xNN-xNN-5') or two (3'-xxNN-xxNN-xxNN-5'). Because the correct targeting logic could not be predicted a priori, all four possibilities were tested systematically.

For each of the 41 vrRNAs from the SUSP1 VIPR clade, the GGY/NN motifs were converted into the four types of nhmmer queries designed to test a different number (0-3 nt) of skipped bases. This was achieved by replacing every conserved GGY trinucleotide with different numbers of wild-card bases (denoted lowercase ‘n’ in HMMER input; distinct from the uppercase N used to describe the variable dinucleotides in vrRNAs). The four replacement strategies were: 1) GGY removed entirely; 2) GGY converted to ‘n’; 3) GGY converted to ‘nn’; and 4) GGY converted to ‘nnn’. For example, a vrRNA with the sequence GGTAAGGTCCGGTGG would yield the queries 1) AACCGG; 2) nAAnCCnGG; 3) nnAAnnCCnnGG; and 4) nnnAAnnnCCnnnGG, respectively.

nhmmer (--max -T 0 --incT 0) was used to search the 164 queries generated using the four different replacement strategies against the E. coli pangenome (4,502 chromosomes and 12,212 plasmids) database created by retrieving all E. coli genomes from NCBI RefSeq. The permissive threshold was necessary because HMMER's scoring is not suited for queries with high degenerate content. Manual testing revealed that each additional wild-card base heavily penalizes scoring such that perfect matches scored progressively lower from NN to xNN to xxNN to xxxNN queries. Manual testing also revealed that longer queries with mismatches can also outscore shorter queries with perfect matches, biasing results toward longer vrRNAs regardless of match quality. For these reasons, neither bit scores nor E-values were used to assess match quality. Instead, hit quality was scored based solely on the fraction of aligned NN positions (hereafter, NN

bioRxiv preprint doi: https://doi.org/10.64898/2026.04.26.720920; this version posted June 25, 2026. The copyright holder for this preprint (which was not certified by peer review) is the author/funder, who has granted bioRxiv a license to display the preprint in perpetuity. It is made available under a CC-BY 4.0 International license.

match) after filtering out all hits that contained internal gaps. Analysis of only gapless hits enforced preservation of the intended skipped base number for each of the four query types.

bioRxiv preprint doi: https://doi.org/10.64898/2026.04.26.720920; this version posted June 25, 2026. The copyright holder for this preprint (which was not certified by peer review) is the author/funder, who has granted bioRxiv a license to display the preprint in perpetuity. It is made available under a CC-BY 4.0 International license.

Analysis of vrRNA natural targets

Hits for the 1 nt skip (NNx) queries with >80% NN matches from the *E. coli* pangenome search were considered natural targets of SUSP1 vrRNAs. For each vrRNA, the top 10 ranking hits were further investigated. In a few instances, the top 10 hits did not converge onto the same locus. In these cases, the most prevalent type of target was kept for further analysis. Of the 20 SUSP1 vrRNAs with identifiable natural targets, 18 converged on a satellite phage that likely parasitizes on SUSP1. The exact sequences of the satellite phage varied across different genomes. The locus diagram summarizing SUSP1 natural vrRNA targets was therefore generated using a consensus approach where satellite phage loci containing top vrRNA hits were aligned to generate a consensus locus. If any member in the alignment contained a hit at a given position, the consensus was annotated as having a hit at that position.

Natural targets of *P. fulva* vrRNAs were identified using the same approach as SUSP1 vrRNA target search. The *P. fulva* Vipr protein was used as a BLASTp query against NCBI NR to identify near-identical VIPR systems (>90% protein identity), resulting in 32 loci that define the *P. fulva* VIPR clade. From these loci, 96 unique vrRNAs were manually identified. The *Pseudomonas* pangenome database (2,609 chromosomes and 1,089 plasmids) was created by retrieving assemblies of all descendant lineages of taxid 286 from NCBI RefSeq. The 96 unique vrRNA guide sequences were converted to 1 nt skip (NNx) queries and searched against this database using nhmmer (-T 6 --incT 6) (the threshold was informed from the SUSP1 vrRNA search). Alignments were filtered with zero-gap-tolerance, and only hits with ≥80% NN match were retained. The consensus target map was generated as described above for SUSP1. Each guide was also searched against its own source contig using nhmmer with identical parameters. No self-targeting hits passed the ≥80% NN match threshold.

bioRxiv preprint doi: https://doi.org/10.64898/2026.04.26.720920; this version posted June 25, 2026. The copyright holder for this preprint (which was not certified by peer review) is the author/funder, who has granted bioRxiv a license to display the preprint in perpetuity. It is made available under a CC-BY 4.0 International license.

To identify potential targets of type III and type V VIPR systems, vrRNAs were manually annotated from the type III and type V subsets of the 664 representative VIPR loci. This resulted in 63 type III and 39 type V vrRNA sequences. Guides were searched against the IMG/VR v4 database (*24*) using the workflow as described above. Of the type III vrRNAs, 44/63 had hits with ≥80% NN match. Of the Type V vrRNAs, 23/39 had hits with ≥80% NN match. As a control, each guide was also searched against its own source contig using the same parameters. No self-targeting hits passed the ≥80% NN match threshold.

To assess sequence preferences beyond the complementary region, sequence contexts flanking the full span of the complementary region of each inferred target site were extracted from the genomic sequence. Sequence logos of the flanking contexts were generated in R from weighted position weight matrices (weighted PWMs), as calculated using $1/n_{\text{sites}}$ as weights, where n_{sites} was the number of sites considered for each vrRNA, to avoid biasing vrRNA with more predicted target sites.

VIPR RNP purification

Protein purification plasmids containing SUSP1 or *P. fulva* VIPR systems were transformed into 10-beta *E. coli* Mix & Go! Competent Cells (Zymo), and plated on LB agar supplemented with ampicillin. Single colonies were inoculated into 50 mL LB media with ampicillin, and grown overnight at 37°C, 180RPM. 25 mL of saturated starter culture was inoculated into 1 L of 2XYT medium containing ampicillin and grown overnight at 37°C, 150RPM. Cultures were cooled on ice for 30 minutes once reaching OD ~0.6, induced with either 500nM crystal violet or 0.2% L-arabinose, and incubated overnight at 16°C, 120RPM. Cells were harvested by centrifugation at 4 kg for 30 minutes, and resuspended in ice-cold lysis/wash buffer (50 mM Tris pH 8.5, 500 mM NaCl, 5 mM $MgCl_2$, 30 mM imidazole, 10% glycerol, 1 mM

bioRxiv preprint doi: https://doi.org/10.64898/2026.04.26.720920; this version posted June 25, 2026. The copyright holder for this preprint (which was not certified by peer review) is the author/funder, who has granted bioRxiv a license to display the preprint in perpetuity. It is made available under a CC-BY 4.0 International license.

TCEP). Cells were sonicated (10 seconds on, 50 seconds off, total processing time 1 minute) in an icebath, and the lysate was clarified by centrifugation at 40'000g for 30 minutes.

The soluble fraction was applied to a Ni-NTA Superflow cartridge (Qiagen) using a peristaltic pump. The loaded column was washed with ≥10 column volumes of lysis buffer, and VIPR RNPs were eluted using five column volumes of elution buffer (50 mM Tris pH 8.5, 500 mM NaCl, 5 mM $MgCl_2$, 300 mM imidazole, 10% glycerol, 1 mM TCEP). Elutions were supplemented with DNase I and TEV protease, and diluted 1:1 with low-salt ion exchange (IEX) buffer (50 mM HEPES pH 6.8, 100 mM KCl, 5 mM $MgCl_2$, 0.1% glycerol, 1 mM TCEP) to bring down salt concentration to ~300 mM. The diluted sample was circulated over a HiTrap Heparin column (Cytiva) using a peristaltic pump for an hour. The loaded column was washed with five column volumes of IEX buffer, and eluted using a linear salt gradient to 1 M KCl over 10 column volumes on an ÄKTA pure chromatography system (Cytiva). The ribonucleoprotein complex typically eluted at ~800 mS/cm conductivity for SUSP1 VIPR, and ~500 mS/cm for *P. fulva* VIPR.

Fractions containing VIPR RNPs (identified by elevated A260/A280 ratios, typically ~1.6) were pooled and concentrated using an Amicon Ultracentrifugal filter, 100 kDa MWCO. Concentrated samples were immediately subjected to size exclusion chromatography (SEC) as the RNPs precipitate upon overnight storage at 4°C. SEC was performed on a Superose 6 Increase column (GE Healthcare) equilibrated in IEX buffer using ÄKTA pure. SUSP1 VIPR RNP eluted as a single peak centered around ~12–14 mL depending on sample concentration, whereas *P. fulva* VIPR RNP eluted as a single peak at ~14 mL. Homogeneity of the final pooled products were ensured using SDS-PAGE with InstantBlue Coomassie Protein Staining, and 6% UREA-PAGE with SYBR gold staining. Samples were flash frozen in liquid nitrogen and stored at −80°C.

bioRxiv preprint doi: https://doi.org/10.64898/2026.04.26.720920; this version posted June 25, 2026. The copyright holder for this preprint (which was not certified by peer review) is the author/funder, who has granted bioRxiv a license to display the preprint in perpetuity. It is made available under a CC-BY 4.0 International license.

DNA substrates for *in vitro* binding assays

Single-stranded DNA oligos were ordered from IDT, and annealed to generate double-stranded DNA (dsDNA) substrates. Equimolar amounts of 5' fluorescein-labeled target strand and unlabeled non-target strand was mixed in DNA Annealing Buffer (10 mM Tris-HCl, pH 8.0; 100 mM NaCl; 1 mM EDTA) to create dsDNA substrates. After heating to 95°C for 5 min, and slowly cooling to 35°C over 45 minutes on a thermocycler, annealed products were quantified by measuring A260 on a NanoDrop spectrophotometer (ThermoFisher Scientific), and concentrations were calculated using manufacturer-provided extinction coefficients. Annealing of dsDNA was confirmed on an 8% native polyacrylamide gel run at 150V at 4°C for 4 hours, and working stocks were diluted to 10 µM in water.

In vitro binding assays

1 µM of purified SUSP1 VIPR RNP was incubated with 1 µM of FAM-labeled target dsDNA and purified salmon sperm competitor DNA (10mg/mL, Invitrogen) in 50mM HEPES (pH 6.8), 100mM KCl, 5mM $MgCL_2$, 0.1% glycerol (v/v), and 1mM TCEP. Competitor DNA concentration ranged from 0x, 1x, 2x, or 10x the mass of the target dsDNA. The reaction was incubated for 1 hour at 37°C, mixed with 2X loading buffer (10mM Tris-HCl (pH 7.5), 50% glycerol (v/v)), and electrophoresed in a 0.5x TBE running buffer at 40V for 90 minutes on a 12% Mini-Protean TGX Precast gel (Bio-Rad). Gels were imaged using an Amershan Typhoon scanner (Cytiva) detecting FAM at 488nm with a Cy2 emission filter.

Green-fluorescent protein (GFP) depletion assays

The VIPR plasmids and target GFP plasmids were introduced into *E. coli* NEB-10B strains (New England Biolabs) via electroporation. Approximately 25 ng of each plasmid was

bioRxiv preprint doi: https://doi.org/10.64898/2026.04.26.720920; this version posted June 25, 2026. The copyright holder for this preprint (which was not certified by peer review) is the author/funder, who has granted bioRxiv a license to display the preprint in perpetuity. It is made available under a CC-BY 4.0 International license.

used in 0.1 mm cuvettes on a Micropulser device (Bio-Rad). Transformed cells were recovered for one-hour, and serially diluted and plated on LB-agar containing ampicillin, kanamycin, and optionally 0.002% L-arabinose. Fluorescence intensity was measured after overnight incubation to assess the depletion of the GFP fluorescence.

Phages strains and culturing

Phage SUSP1 was provided by Sankar Adhya. SUSP1 was propagated on *E. coli* BW25113 in LB at 37°C. *E. coli* MG1655 carrying a lysogen of phage $\lambda_{bor::kan}$ (henceforth, *E. coli* MG1655::$\lambda_{bor::kan}$), was provided by Rodolphe Barrangou. *E. coli* MG1655::$\lambda_{bor::kan}$ was grown in LB + kanamycin at 30ºC to prevent prophage induction. A virulent variant of phage Lambda (λvir), provided by Luciano Marraffini. λvir was scaled on *E. coli* BW25113 in LB at 37°C using the webbed plate lysis method. Cleared and filtered lysates of λvir were stored at 4ºC, with their titres determined via plaque assays on empty vector-carrying NEB 10-beta cells.

Plaque assays

Plaque assays were performed using the double agar overlay method. 100 µL of saturated overnight cultures carrying either a GFP- or λ-targeting vrRNA and *vipr* gene were added to 5 mL of molten LB agar (0.7% agar, ~50°C; henceforth, "top agar") supplemented with antibiotics, and optionally inducer. For plaque assays that assessed the antiviral activity of the vrRNA-*vipr* operon against λvir-cro targets, top agar was supplemented with crystal violet at a final concentration of 100 nM to promote expression of the targeting complex. For plaque assays that assessed the combined antiviral activity of the vrRNA-*vipr* operon and the *vap1* gene, no additional inducers were added to the top agar because the vrRNA-*vipr* alone restricted λvir to near limit of detection

bioRxiv preprint doi: https://doi.org/10.64898/2026.04.26.720920; this version posted June 25, 2026. The copyright holder for this preprint (which was not certified by peer review) is the author/funder, who has granted bioRxiv a license to display the preprint in perpetuity. It is made available under a CC-BY 4.0 International license.

when induced. The supplemented top agar was poured over 1.5% LB agar plates with antibiotics, and was allowed to cool to room temperature and solidify. Tenfold phage dilutions were prepared in SM buffer (Teknova), then 2.5 µL of this dilution series was spotted onto the top agar. After drying under sterile conditions, plates were incubated at 37ºC overnight. Plaque assays were performed in biological triplicate.

Liquid infection assays

Saturated overnight cultures carrying either a GFP- or λ-targeting vrRNA, alongside the SUSP1 *vipr* gene, were seeded in a microplate (Corning) at ~8e6 CFU per well in 200 µL of LB supplemented with carbenicillin and 100nM crystal violet. Tenfold dilutions of λvir in SM buffer were added to cells, and growth was monitored by measurement of OD600 every 5 minutes while shaking at 800 RPM (double orbital) at 37ºC overnight on a 96-well plate reader (Biotek Cytation 5).

VIPR-targeted prophage induction assays

Overnight cultures of *E. coli* MG1655::$\lambda_{bor::kan}$ were diluted 1:50 in fresh LB + kanamycin media, and grown until mid-log phase. Cells were pelleted by centrifugation at 4 kg for 10 minutes, and washed five times with ice-cold 10% glycerol. Washed cells were electroporated with 750 ng of a plasmid encoding either a GFP- or cI-targeting vrRNA and *vipr* gene. The electroporated cells were recovered in LB + kanamycin & carbenicillin, and grown overnight at 30ºC, 180RPM. To harvest induced phages, cells and debris were pelleted by centrifugation at 4 kg for 10 minutes. The supernatant fraction containing induced phages was harvested, and tenfold dilutions were prepared in SM buffer before spotting onto top agar lawns of MG1655. After drying under sterile conditions, plates were incubated at 37ºC overnight.

bioRxiv preprint doi: https://doi.org/10.64898/2026.04.26.720920; this version posted June 25, 2026. The copyright holder for this preprint (which was not certified by peer review) is the author/funder, who has granted bioRxiv a license to display the preprint in perpetuity. It is made available under a CC-BY 4.0 International license.

Figure S1

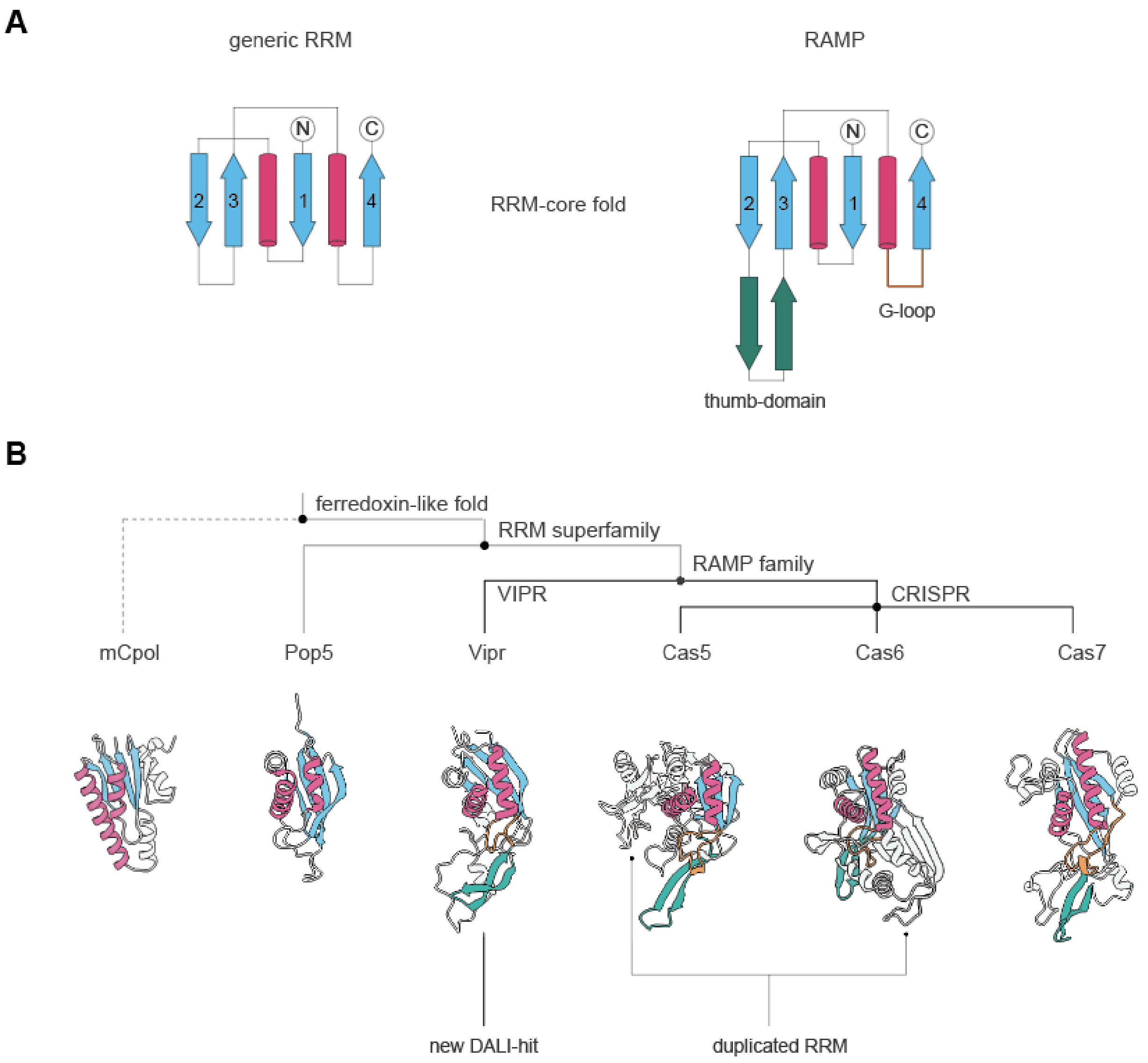

Fig. S1. Structural comparison of RAMPs and their relatives. (A) Secondary structure comparisons of generic RRM to RAMPs. **(B)** Dendrogram of the ferredoxin-like fold, RRM superfamily, and RAMP family (top), and color coded structures of a representative from each group (bottom).

bioRxiv preprint doi: https://doi.org/10.64898/2026.04.26.720920; this version posted June 25, 2026. The copyright holder for this preprint (which was not certified by peer review) is the author/funder, who has granted bioRxiv a license to display the preprint in perpetuity. It is made available under a CC-BY 4.0 International license.

Figure S2

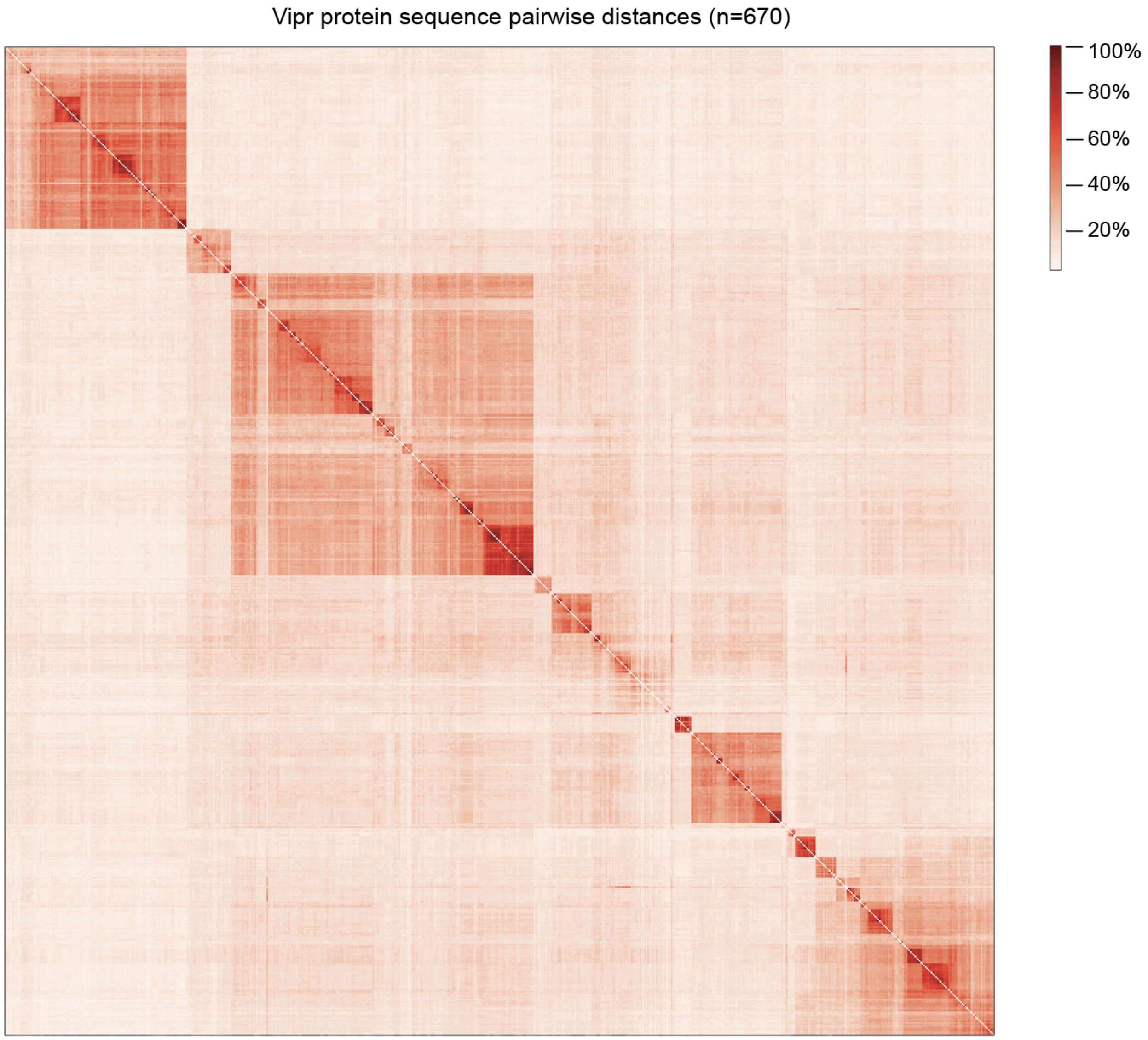

Fig. S2. Heatmap of Vipr protein sequence comparisons. Depicts pairwise % sequence identity of 670 representative Vipr protein sequences identified in this study.

bioRxiv preprint doi: https://doi.org/10.64898/2026.04.26.720920; this version posted June 25, 2026. The copyright holder for this preprint (which was not certified by peer review) is the author/funder, who has granted bioRxiv a license to display the preprint in perpetuity. It is made available under a CC-BY 4.0 International license.

Figure S3

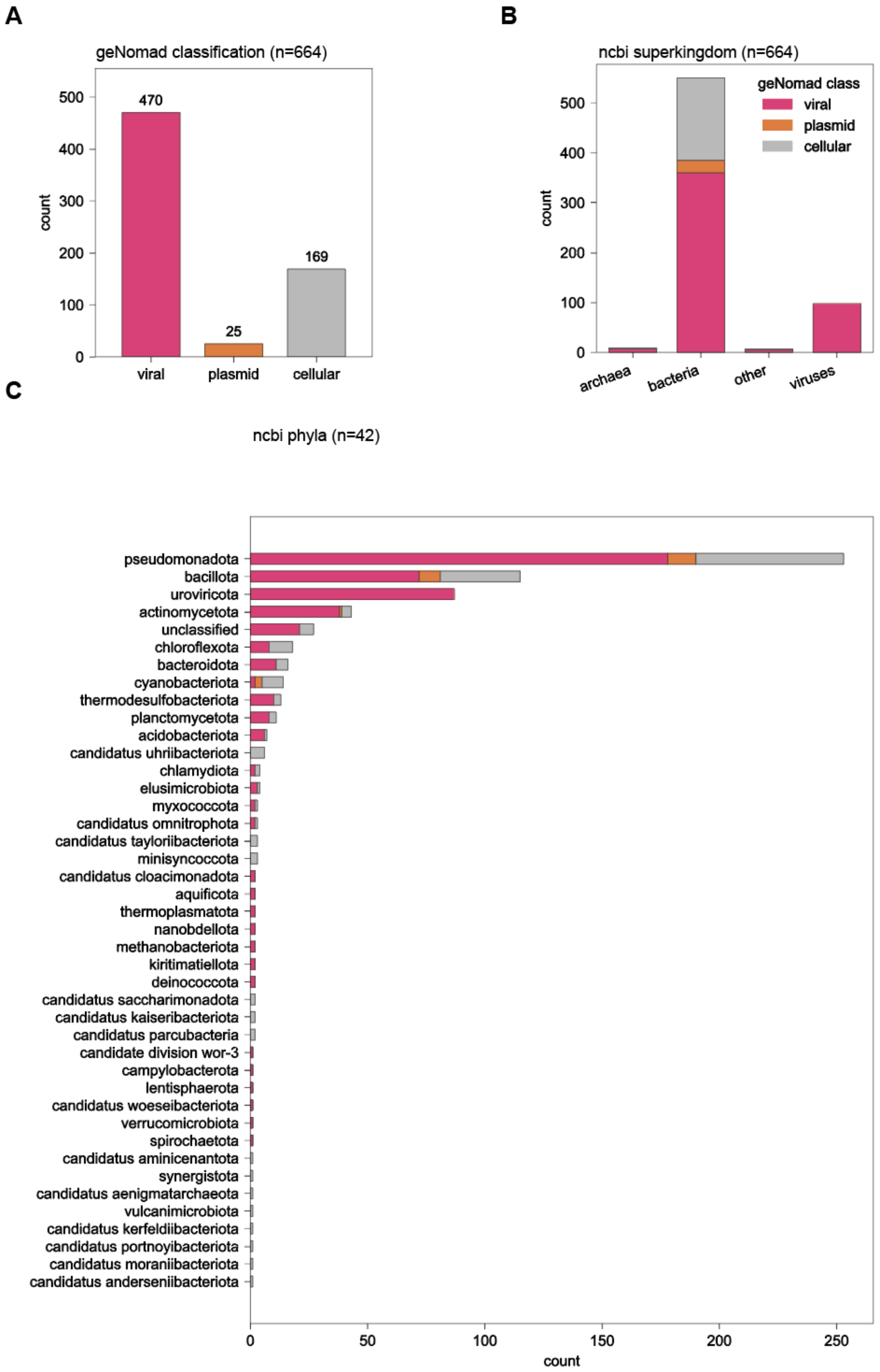

Fig. S3. Taxonomic distribution of VIPR. (A) geNomad classification of VIPR systems occurring across different genome types. **(B)** Classification of VIPR across superkingdoms. **(C)** Detailed taxonomic classification of VIPR based on phyla.

bioRxiv preprint doi: https://doi.org/10.64898/2026.04.26.720920; this version posted June 25, 2026. The copyright holder for this preprint (which was not certified by peer review) is the author/funder, who has granted bioRxiv a license to display the preprint in perpetuity. It is made available under a CC-BY 4.0 International license.

Figure S4

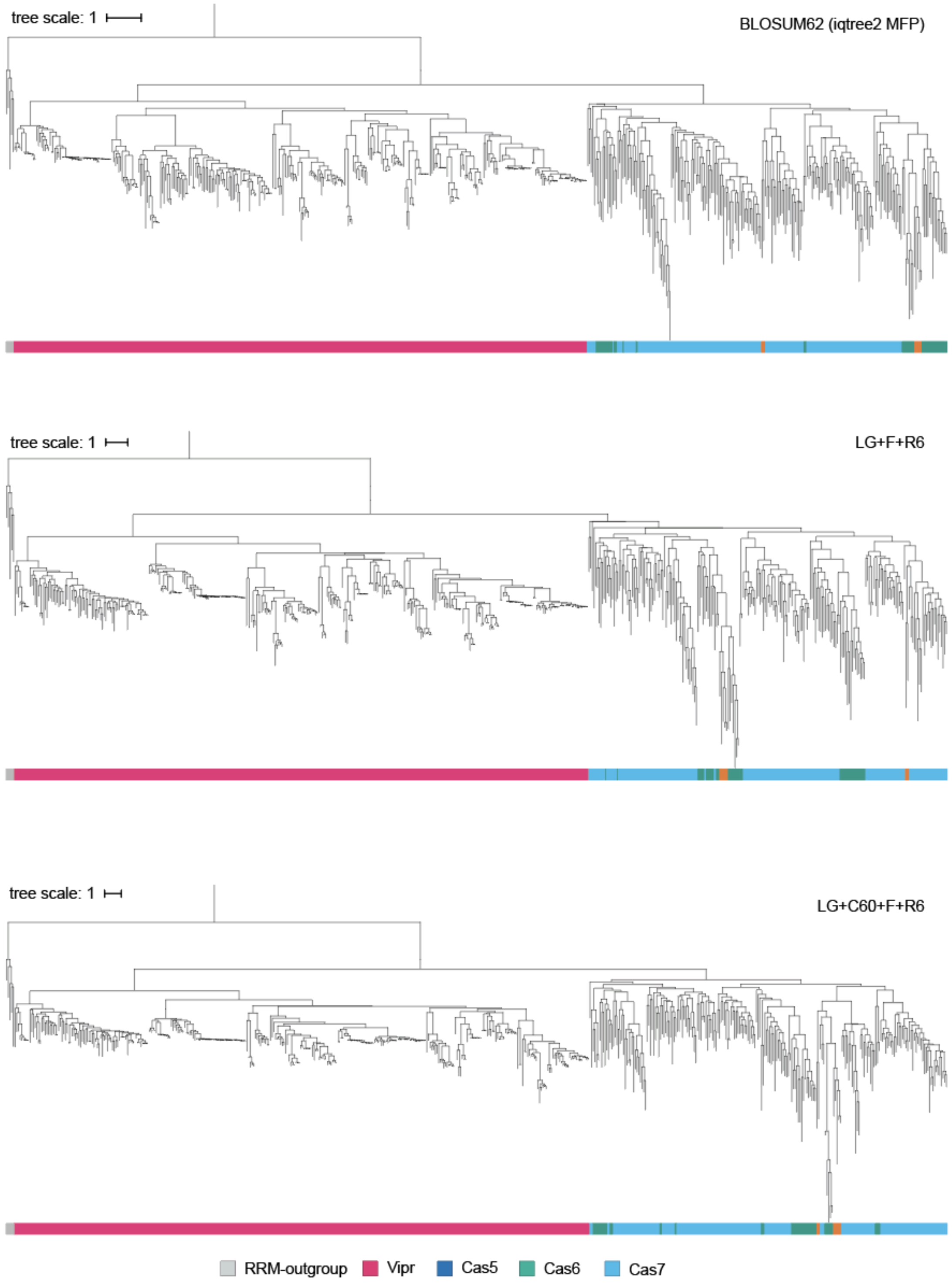

Fig. S4. Phylogenetic trees of RAMPs across different evolutionary models. Maximum-likelihood phylogenetic trees of RAMP genes. Trees were constructed with three substitution models: MFP (BLOSUM62), LG+F+R6, and LG+C60+F+R6. RRM-outgroup (Pop5/non-RAMP RRM). Sequences, alignment, and trees are provided in data S1.

bioRxiv preprint doi: https://doi.org/10.64898/2026.04.26.720920; this version posted June 25, 2026. The copyright holder for this preprint (which was not certified by peer review) is the author/funder, who has granted bioRxiv a license to display the preprint in perpetuity. It is made available under a CC-BY 4.0 International license.

Figure S5

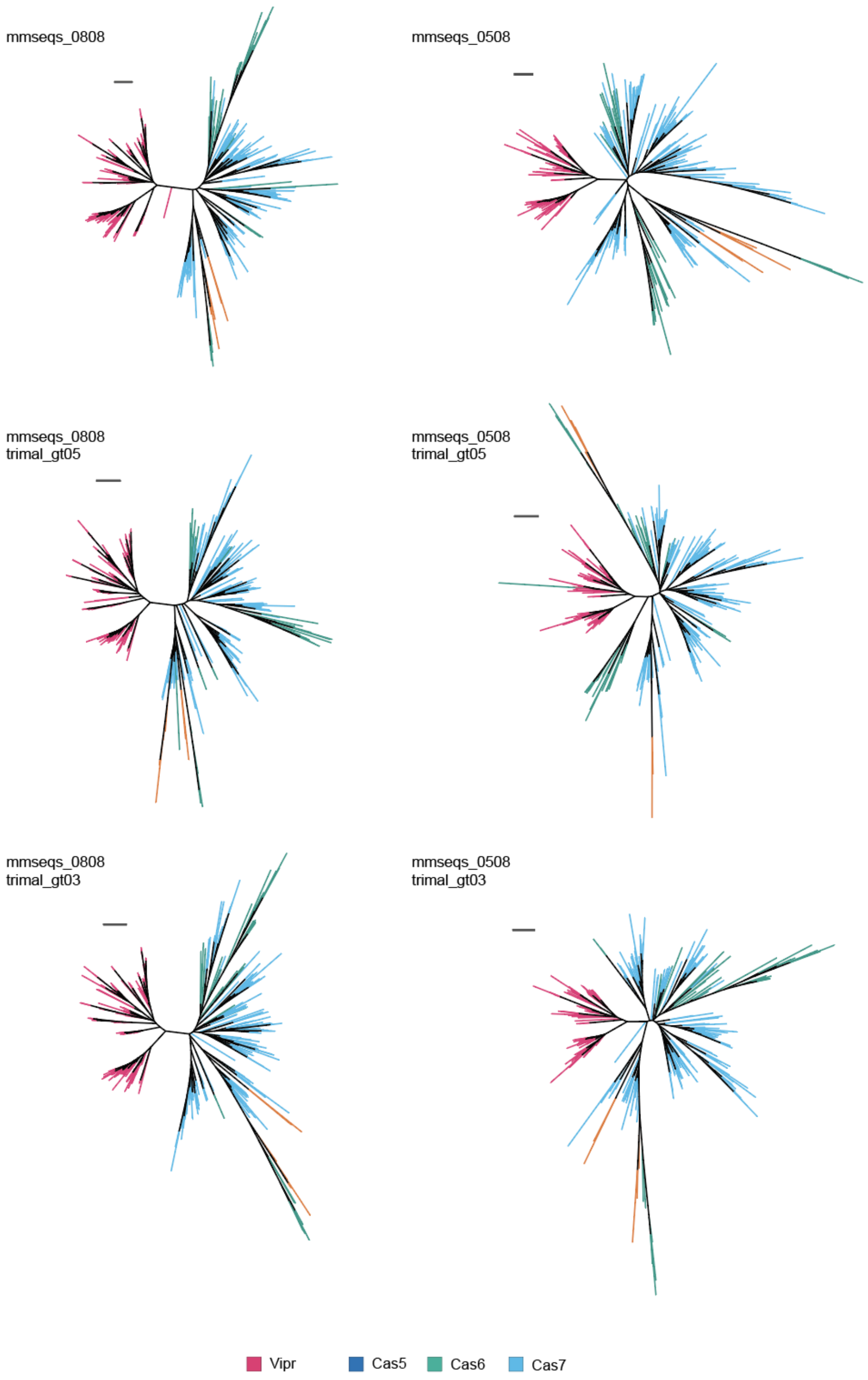

Fig. S5. Phylogenetic trees of RAMPs across clustering and trimming parameters. Unrooted trees of RAMP genes. The trimmed alignment from Fig. S4, comprising the conserved RRM, thumb, and G-loop regions, was clustered at 50% or 80% identity at 80% coverage and trimmed with gap thresholds of 0.3 or 0.5. Trees were inferred from each combination independently. Scale bar = 1. Sequences, alignments, and trees are provided in data S1.

bioRxiv preprint doi: https://doi.org/10.64898/2026.04.26.720920; this version posted June 25, 2026. The copyright holder for this preprint (which was not certified by peer review) is the author/funder, who has granted bioRxiv a license to display the preprint in perpetuity. It is made available under a CC-BY 4.0 International license.

Figure S6

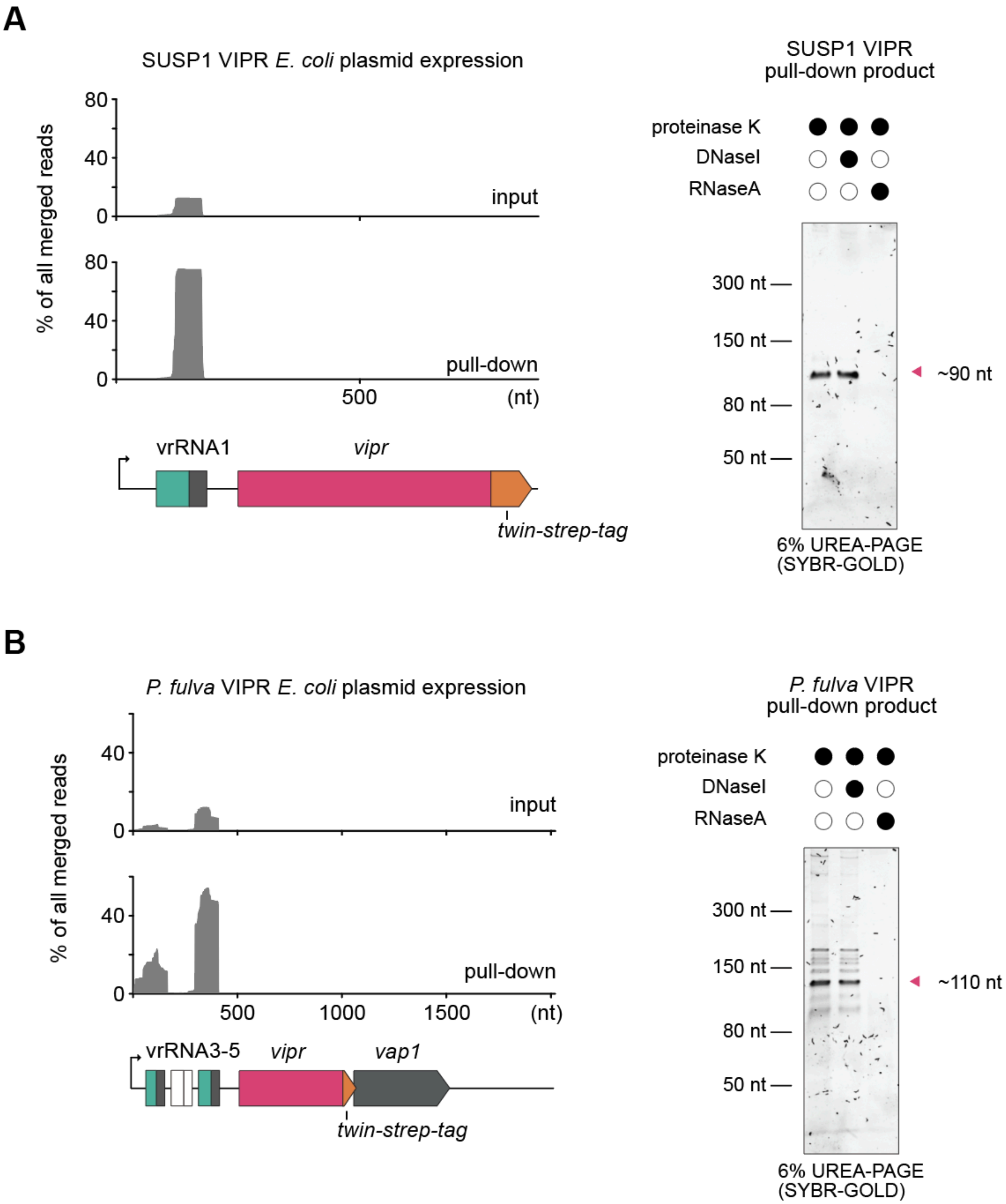

Fig. S6. Vipr RNA pull-down using heterologously expressed VIPR in *E. coli*. sRNA-seq and denaturing PAGE visualization of purified RNP from **(A)** SUSP1 VIPR and **(B)** *P. fulva* VIPR.

bioRxiv preprint doi: https://doi.org/10.64898/2026.04.26.720920; this version posted June 25, 2026. The copyright holder for this preprint (which was not certified by peer review) is the author/funder, who has granted bioRxiv a license to display the preprint in perpetuity. It is made available under a CC-BY 4.0 International license.

Figure S7

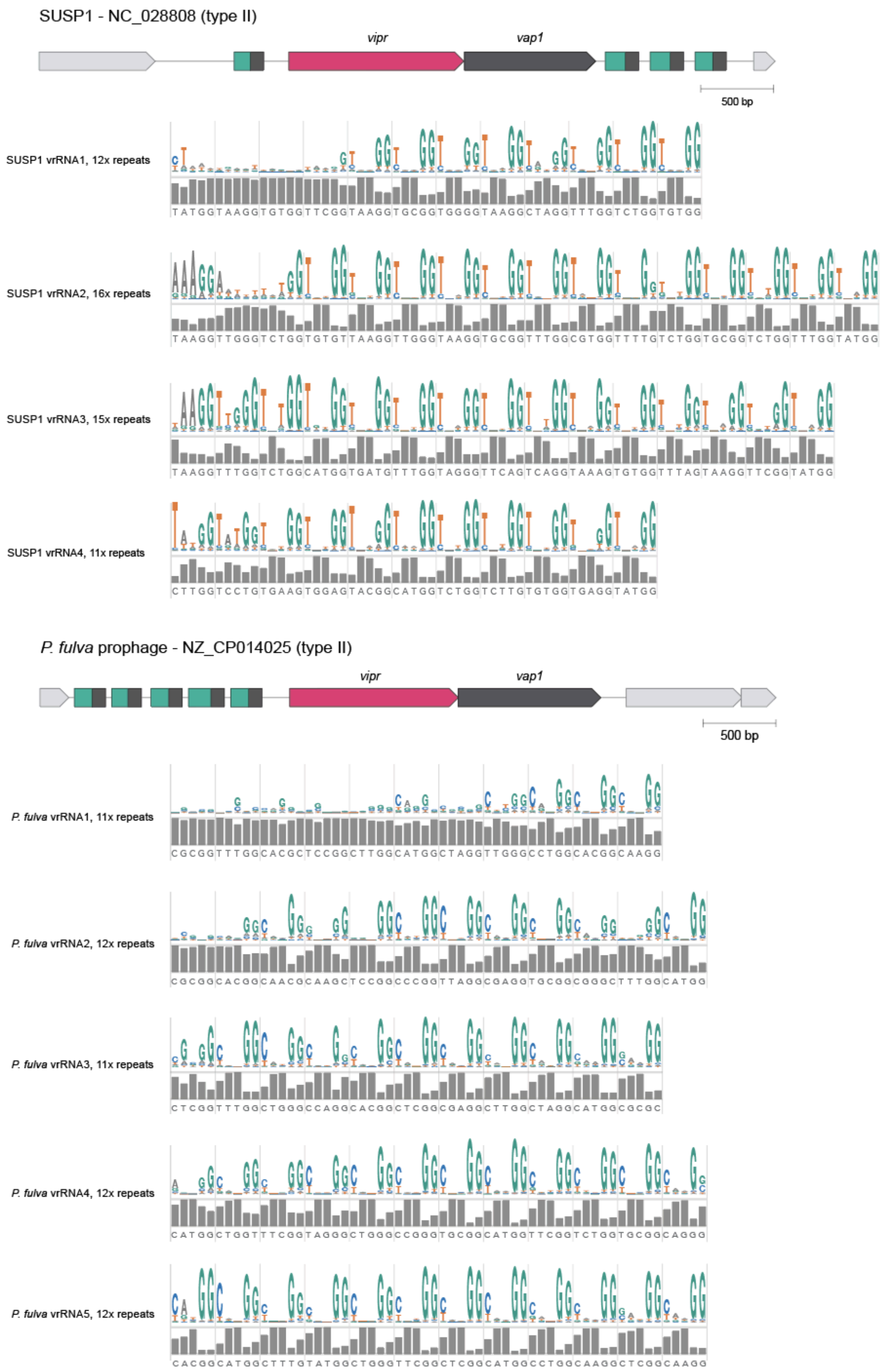

Fig. S7. Evo2-based sequence logos and entropy plots of vrRNA tandem repeats. Tandem repeat sequences from SUSP1 and *P. fulva* prophage vrRNAs were analyzed with Evo2 to generate sequence logos and per-position entropy plots. Each row corresponds to a distinct vrRNA repeat set, with the number of repeats indicated at left. Vertical lines demarcate YNNGG pentanucleotide repeat units.

bioRxiv preprint doi: https://doi.org/10.64898/2026.04.26.720920; this version posted June 25, 2026. The copyright holder for this preprint (which was not certified by peer review) is the author/funder, who has granted bioRxiv a license to display the preprint in perpetuity. It is made available under a CC-BY 4.0 International license.

Figure S8

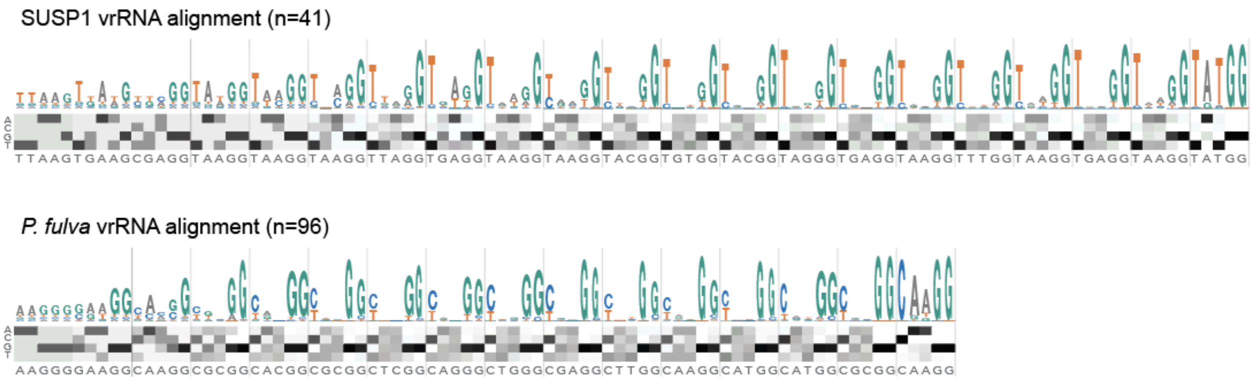

Fig. S8. Sequence conservation across SUSP1 and *P. fulva* vrRNA alignments. Ungapped alignments of SUSP1 vrRNAs and *P. fulva* vrRNAs were used for generating sequence logo plots. Below each logo plot, a heatmap displays the per-position nucleotide composition of the alignment. Each column corresponds to an alignment position, and rows 1–4 represent the smoothed per-position frequencies of A, C, G, and T (refer to Methods for details). Scale white to black (frequency 0 to 100%).

bioRxiv preprint doi: https://doi.org/10.64898/2026.04.26.720920; this version posted June 25, 2026. The copyright holder for this preprint (which was not certified by peer review) is the author/funder, who has granted bioRxiv a license to display the preprint in perpetuity. It is made available under a CC-BY 4.0 International license.

Figure S9

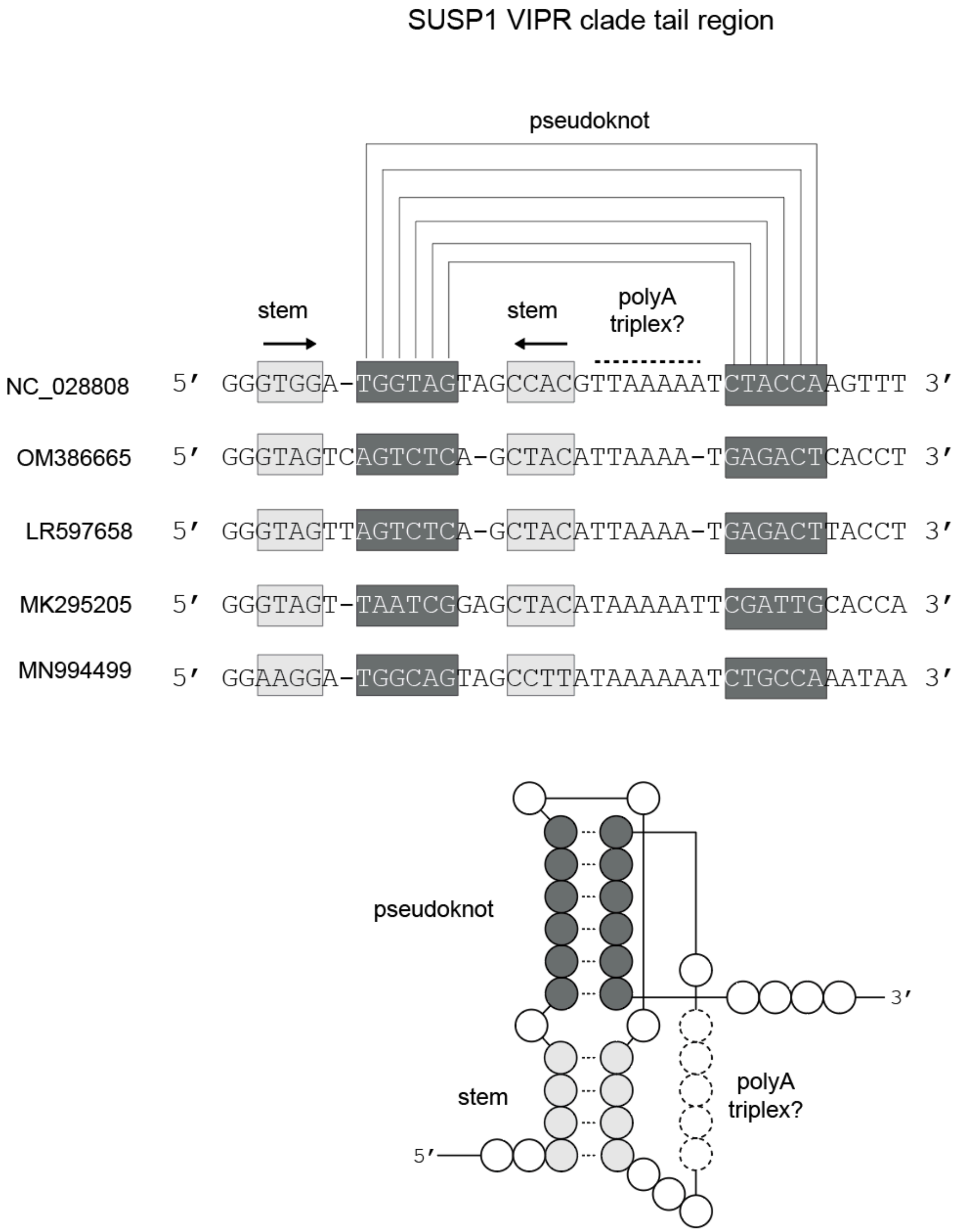

Fig. S9. Covariation analysis and predicted structure for SUSP1 vrRNA tail region. Multiple sequence alignment of five vrRNA tail sequences annotated with predicted structural features (top), and predicted consensus secondary structure diagram (bottom). Arrows depict the orientation of the hairpin half. Brackets depict base pairs in the pseudoknot region.

bioRxiv preprint doi: https://doi.org/10.64898/2026.04.26.720920; this version posted June 25, 2026. The copyright holder for this preprint (which was not certified by peer review) is the author/funder, who has granted bioRxiv a license to display the preprint in perpetuity. It is made available under a CC-BY 4.0 International license.

Figure S10

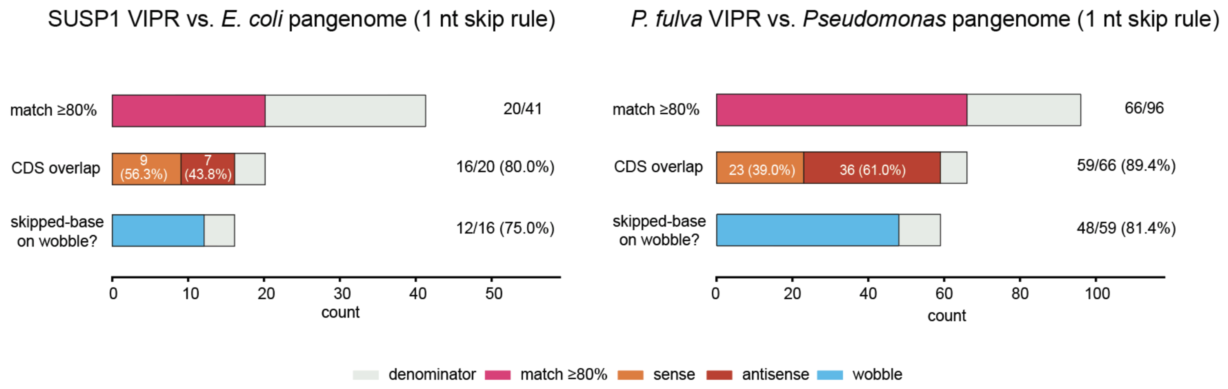

Fig. S10. Coding sequence overlap and codon-position bias of VIPR targets. Summary of 1 nt skip rule search for SUSP1 VIPR against the *E. coli* pangenome, and *P. fulva* VIPR against the *Pseudomonas* pangenome. Top bars show fraction of targets with match scores >80%. Middle bars show fraction of targets to coding sequences (CDS) on the sense or antisense strand. Bottom bars show how often the skip base aligns to the third (wobble) codon position among CDS matches. Overall counts are indicated at right of each bar, whereas sense and antisense overlaps to CDS are indicated within the middle bar.

bioRxiv preprint doi: https://doi.org/10.64898/2026.04.26.720920; this version posted June 25, 2026. The copyright holder for this preprint (which was not certified by peer review) is the author/funder, who has granted bioRxiv a license to display the preprint in perpetuity. It is made available under a CC-BY 4.0 International license.

Figure S11

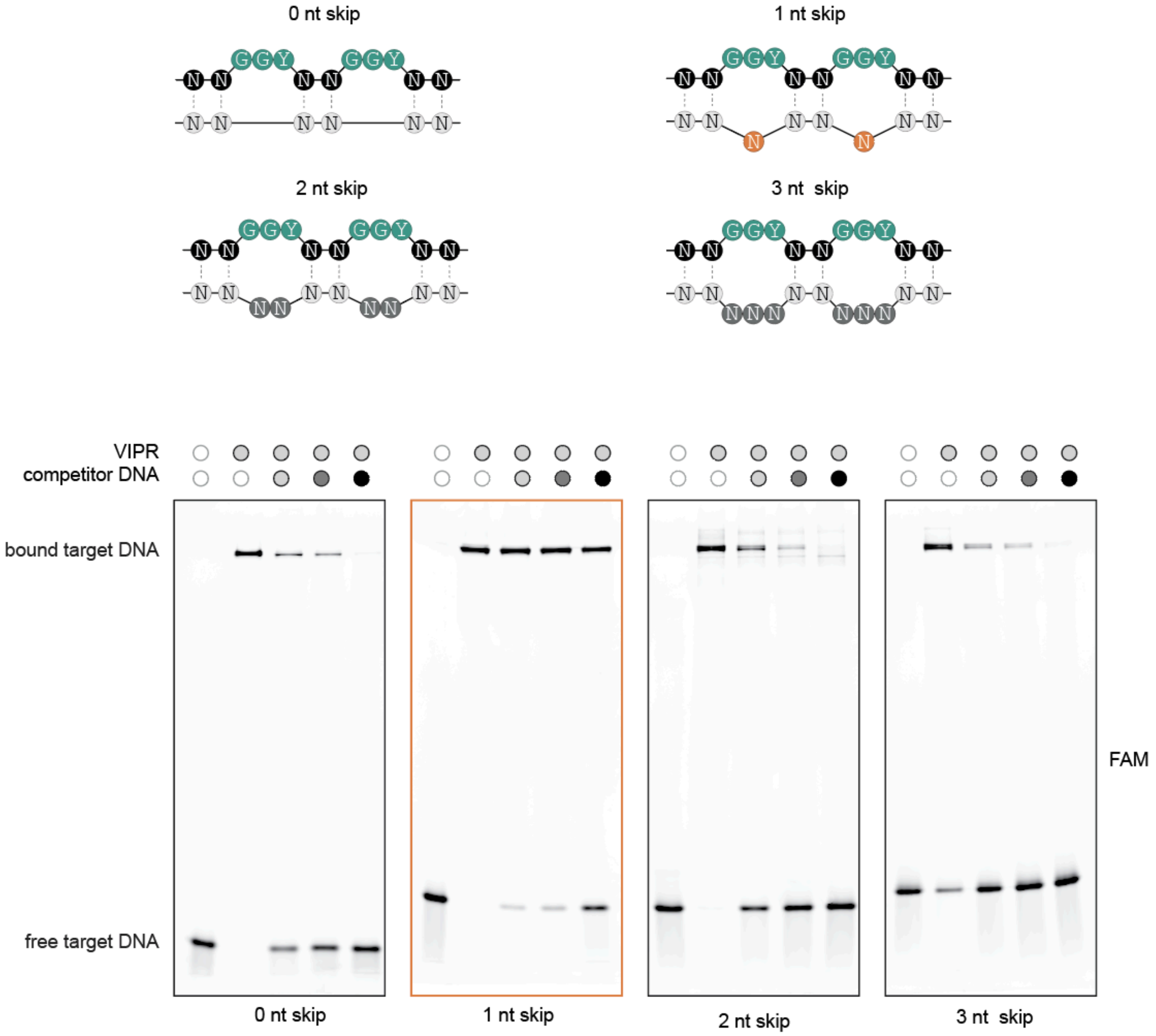

Fig. S11. ***In vitro* competitive binding assay with SUSP1 VIPR**. Diagrams of the different skip rule patterns tested (top). Fluorescein (FAM) channel imaging of 12% native PAGE gel depicting SUSP1 VIPR RNP binding to different skip rule patterned DNA target in a competitive binding assay. Salmon sperm DNA was supplied as a competitor substrate at a ratio of 0, 1, 2, and 10 fold excess by weight compared to the FAM labeled substrate.

bioRxiv preprint doi: https://doi.org/10.64898/2026.04.26.720920; this version posted June 25, 2026. The copyright holder for this preprint (which was not certified by peer review) is the author/funder, who has granted bioRxiv a license to display the preprint in perpetuity. It is made available under a CC-BY 4.0 International license.

Figure S12

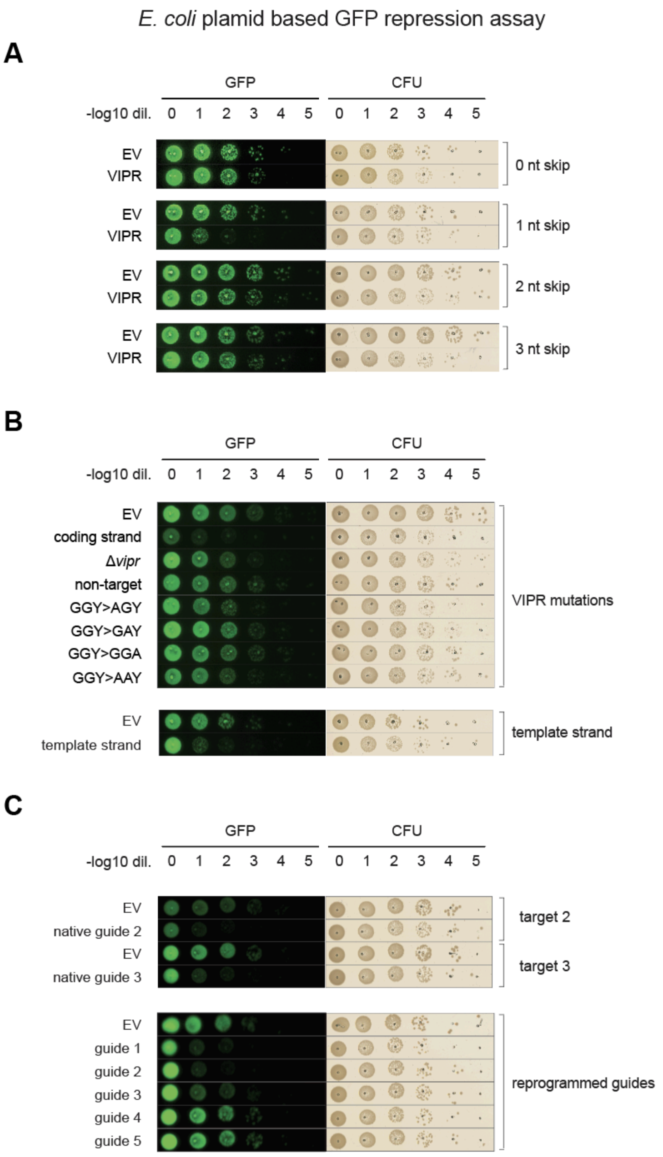

Fig. S12. GFP repression assay. GFP fluorescence (left) and brightfield imaging (right) of serial dilution spot plates of *E. coli* co-transformed with VIPR and GFP target plasmids. **(A)** Testing of 0-3 nt skip rule recognition. Target plasmids were constructed with putative target sites inserted between the promoter and GFP CDS following 0-3 nt skip patterns. The empty vector (EV) has the same backbone as the VIPR effector plasmid, but lacks the VIPR system. **(B)** VIPR mutations tested for minimal component required for GFP repression. "Coding strand" denotes the positive control, in which the VIPR effector targets the coding strand immediately upstream of the GFP CDS. Constructs tested include: ΔVipr, non-targeting guide control (NN directed to unrelated sequence), GGY substitutions (GGY>AGY, GGY>GAY, GGY>GGA, GGY>AAY), and template strand targeting, in which the reverse complement of the native target site was installed at the analogous position. **(C)** Native guides 2 and 3 tested against cognate target 2 and target 3 sites, respectively. Reprogrammed guides (guides 1–5) tested against a GFP target plasmid lacking any inserted predicted target sites.

bioRxiv preprint doi: https://doi.org/10.64898/2026.04.26.720920; this version posted June 25, 2026. The copyright holder for this preprint (which was not certified by peer review) is the author/funder, who has granted bioRxiv a license to display the preprint in perpetuity. It is made available under a CC-BY 4.0 International license.

Figure S13

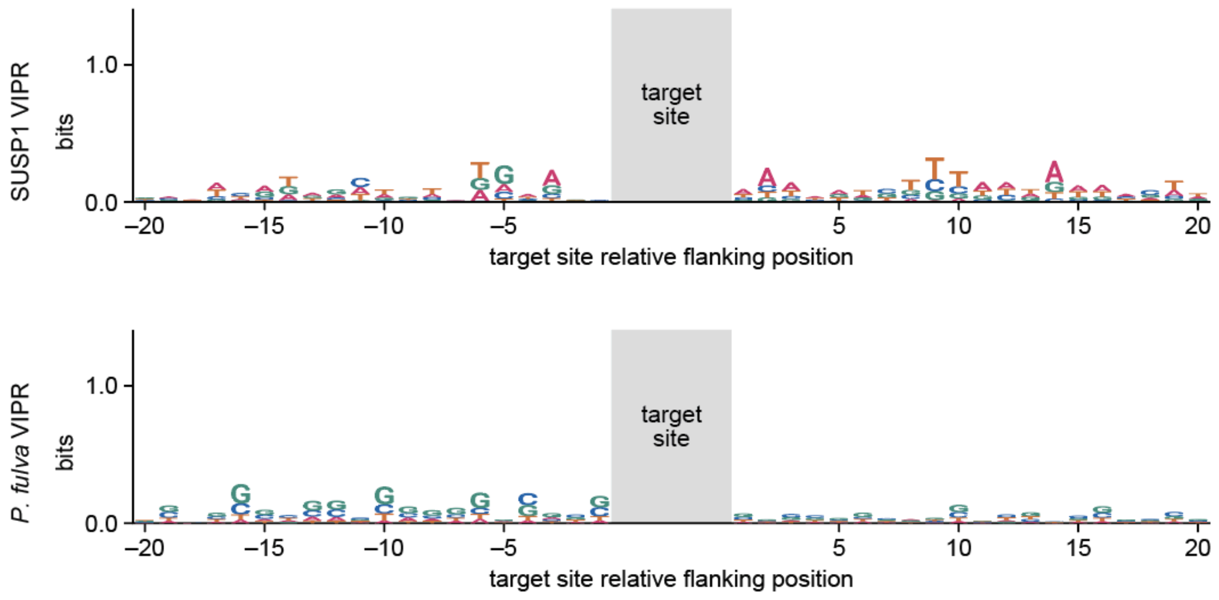

Fig. S13. Sequence logo analysis of sequences flanking vrRNAs target sites. Sequence conservation (bits) at positions flanking the pairing site for SUSP1 (top) and *P. fulva* (bottom) vrRNA target sites. Sequences were extracted from high-confidence genomic matches and aligned relative to the pairing site (gray shading). Low information content is observed at all flanking positions for both VIPR clades.

bioRxiv preprint doi: https://doi.org/10.64898/2026.04.26.720920; this version posted June 25, 2026. The copyright holder for this preprint (which was not certified by peer review) is the author/funder, who has granted bioRxiv a license to display the preprint in perpetuity. It is made available under a CC-BY 4.0 International license.

Figure S14

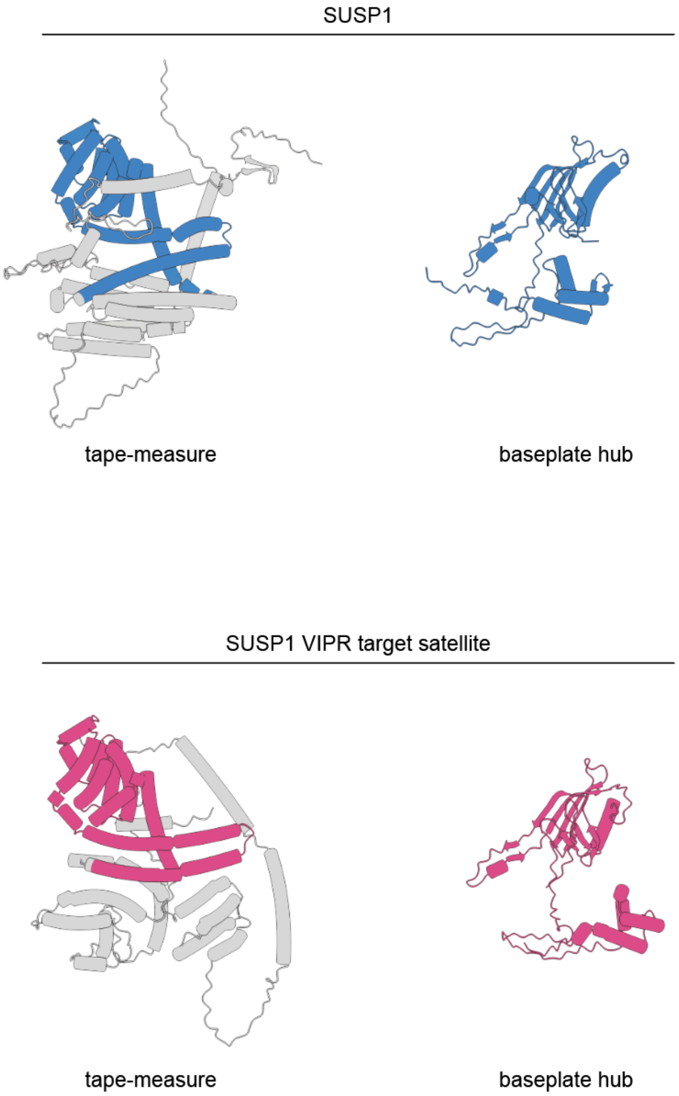

Fig. S14. Homology of structural genes for SUSP1 and the VIPR targeted satellite. AlphaFold3 models of phage tape-measure (left column) and baseplate hub (right column) proteins from SUSP1 (YP_009199406.1, YP_009199407.1) and the satellite phage targeted by the SUSP1 VIPR system (WP_096972664.1, WP_096972663.1). Tape-measures share 26% ID, and baseplates share 72% ID. Blue and magenta highlight the conserved structural elements of SUSP1 and the satellite phage respectively.

bioRxiv preprint doi: https://doi.org/10.64898/2026.04.26.720920; this version posted June 25, 2026. The copyright holder for this preprint (which was not certified by peer review) is the author/funder, who has granted bioRxiv a license to display the preprint in perpetuity. It is made available under a CC-BY 4.0 International license.

Figure S15

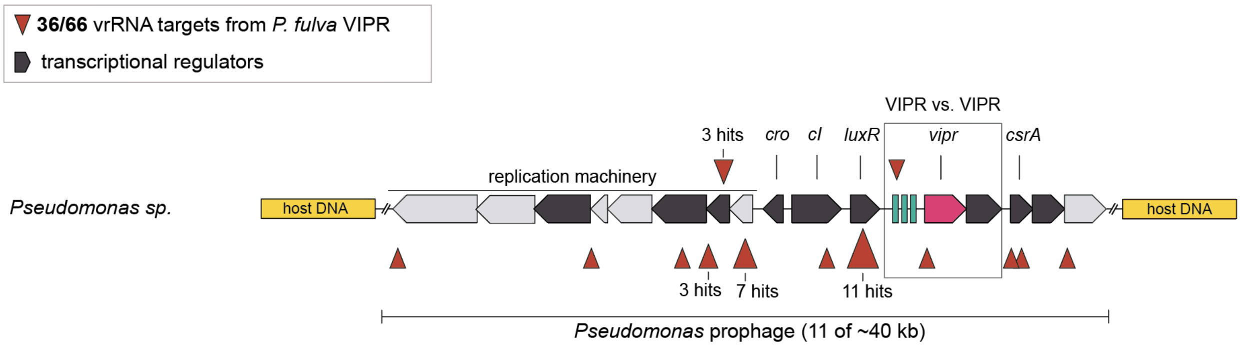

Fig. S15. Locus diagram of *P. fulva* VIPR clade vrRNA targets.

bioRxiv preprint doi: https://doi.org/10.64898/2026.04.26.720920; this version posted June 25, 2026. The copyright holder for this preprint (which was not certified by peer review) is the author/funder, who has granted bioRxiv a license to display the preprint in perpetuity. It is made available under a CC-BY 4.0 International license.

Figure S16

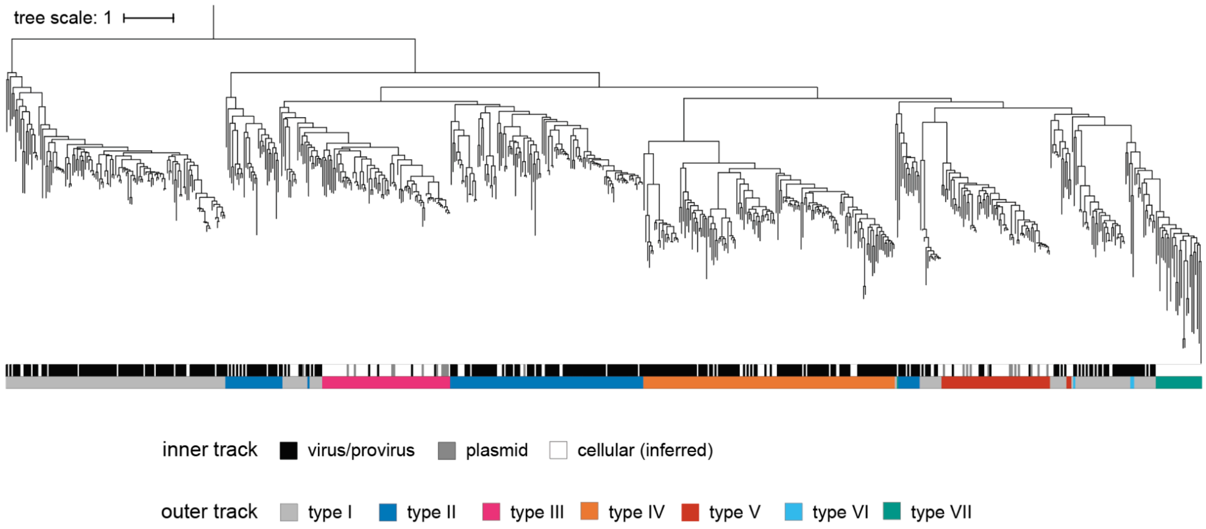

Fig. S16. Maximum-likelihood phylogenetic tree of Vipr proteins. Inner track denotes genome type classification. Outer track denotes VIPR type classification (types I-VII).

Figure S17

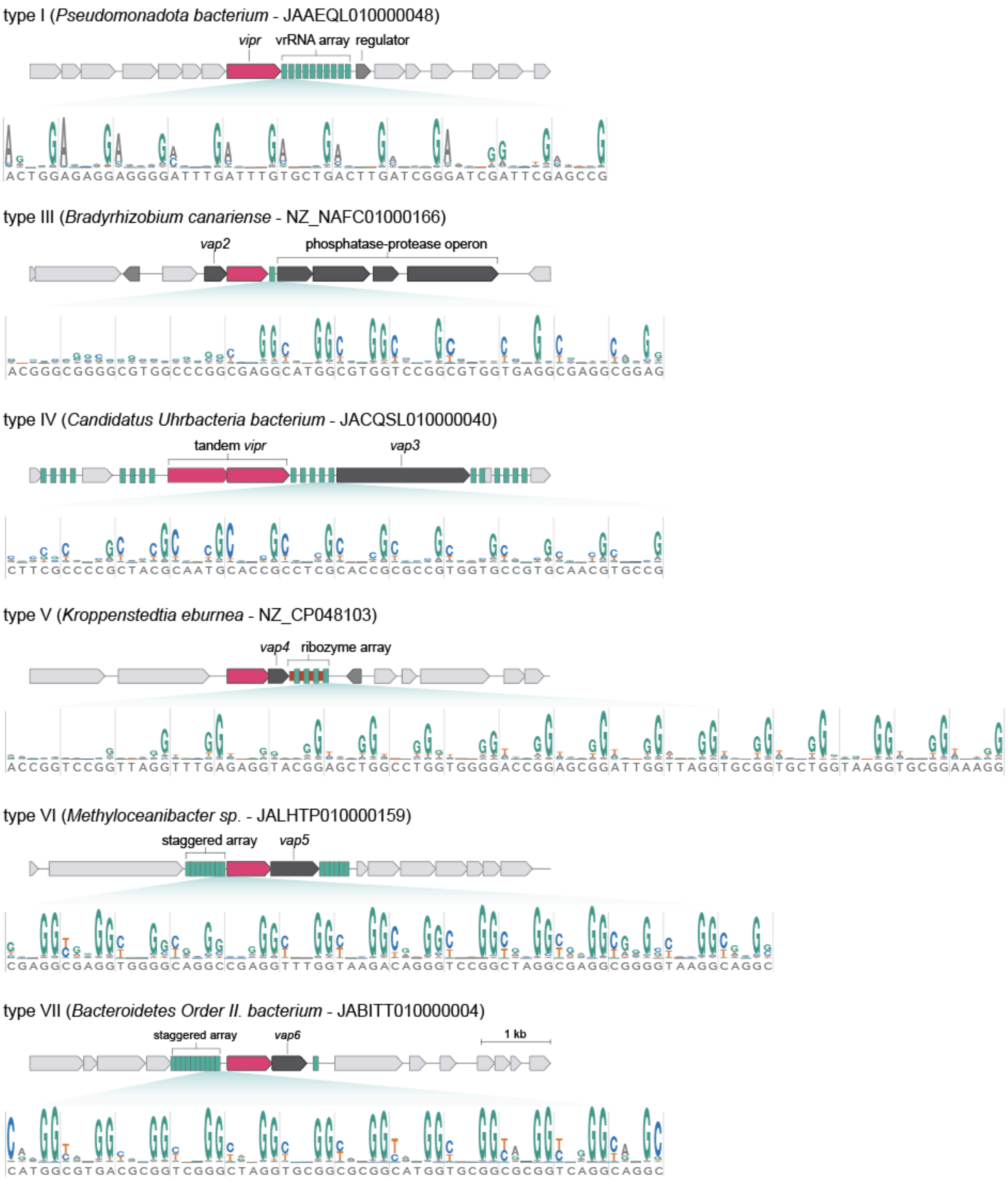

Fig. S17. Locus diagrams and vrRNA Evo2 conservation logos for VIPR types I, III, IV, V, VI, and VII. For each type, a representative genomic locus (top) is shown alongside an Evo2-based sequence logo of the vrRNA tandem repeat region illustrating pentanucleotide motif across types (bottom). Type II (exemplified by SUSP1 and *P. fulva* VIPR systems) is shown in Figs. 2 and S6.

bioRxiv preprint doi: https://doi.org/10.64898/2026.04.26.720920; this version posted June 25, 2026. The copyright holder for this preprint (which was not certified by peer review) is the author/funder, who has granted bioRxiv a license to display the preprint in perpetuity. It is made available under a CC-BY 4.0 International license.

Figure S18

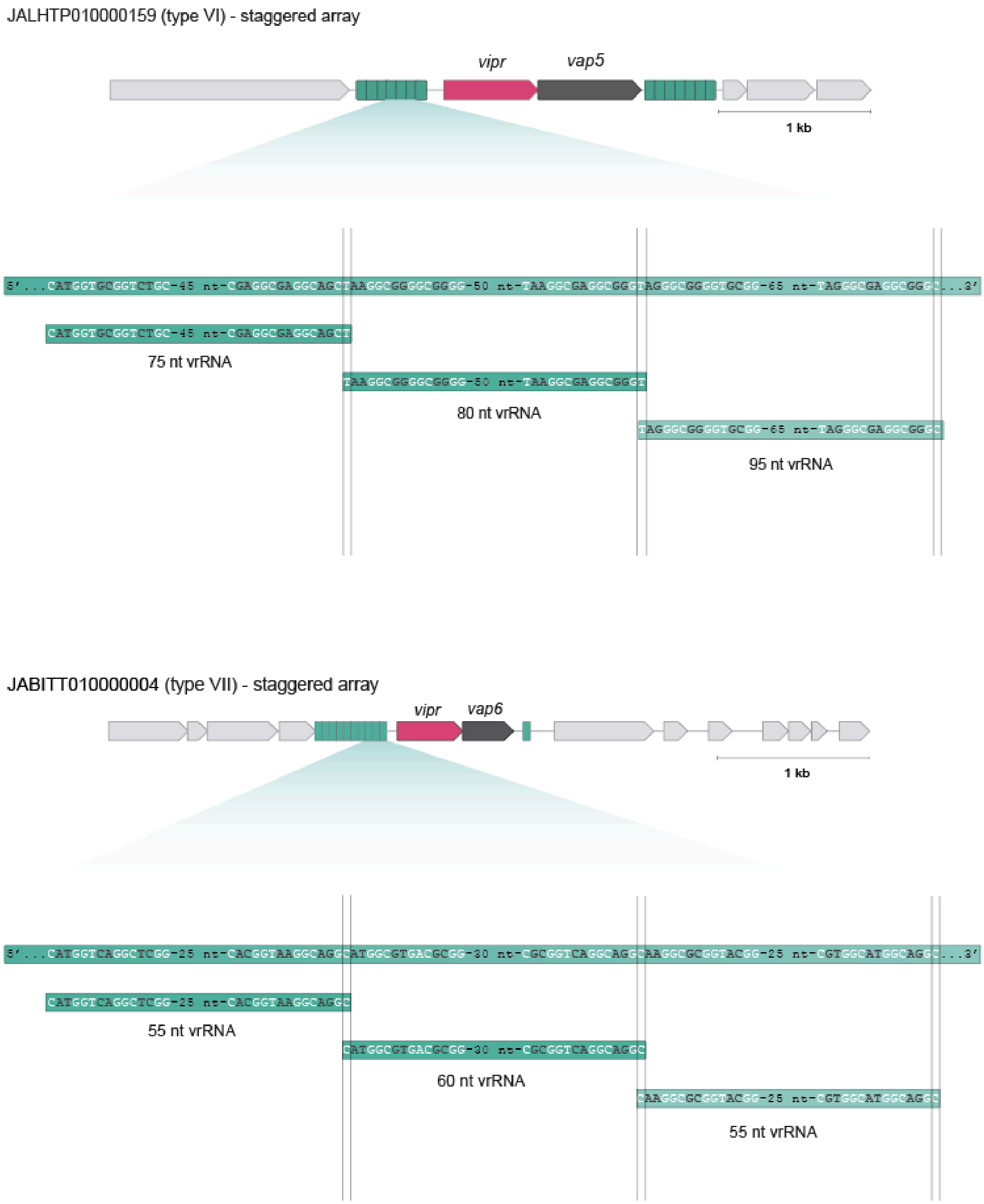

Fig. S18. Staggered vrRNA arrays. A representative genomic locus (top) is shown alongside a zoom in depicting organization of staggered vrRNA arrays (bottom). As depicted in the zoom in diagram, individual vrRNAs are derived from 1 nt overlapping positions within the array. The vertical gray lines mark the boundaries of individual processed vrRNAs, highlighting how the end of one vrRNA marks the start of another vrRNA.

bioRxiv preprint doi: https://doi.org/10.64898/2026.04.26.720920; this version posted June 25, 2026. The copyright holder for this preprint (which was not certified by peer review) is the author/funder, who has granted bioRxiv a license to display the preprint in perpetuity. It is made available under a CC-BY 4.0 International license.

Figure S19

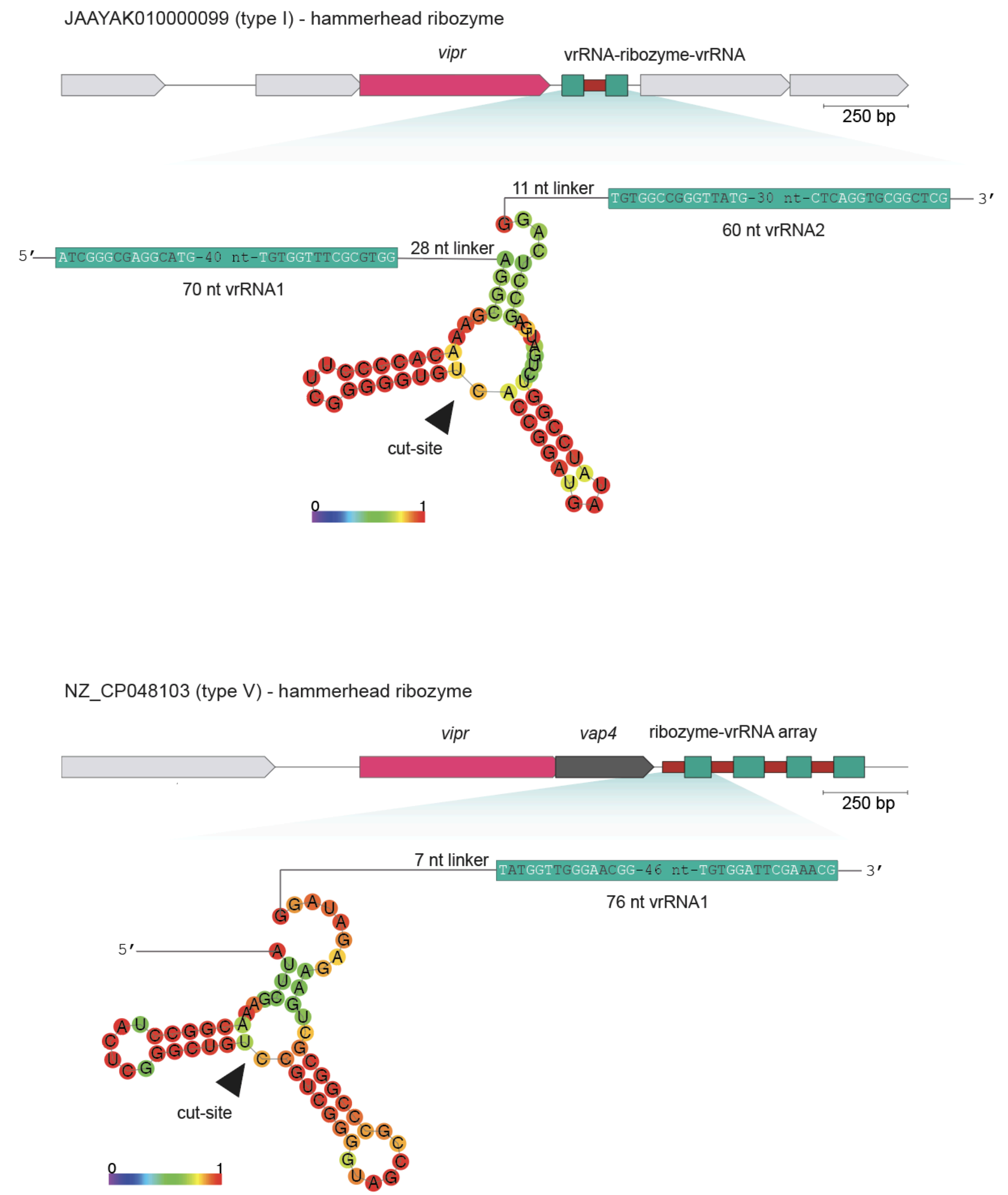

Fig. S19. Ribozyme-vrRNA arrays. A representative genomic locus (top) is shown alongside a zoom in depicting ribozyme-vrRNA architectures (bottom). Predicted hammerhead ribozyme secondary structures are shown between adjacent vrRNA segments, with the inferred cleavage site indicated by an arrowhead. Example loci from type I and type V systems are shown.

bioRxiv preprint doi: https://doi.org/10.64898/2026.04.26.720920; this version posted June 25, 2026. The copyright holder for this preprint (which was not certified by peer review) is the author/funder, who has granted bioRxiv a license to display the preprint in perpetuity. It is made available under a CC-BY 4.0 International license.

Figure S20

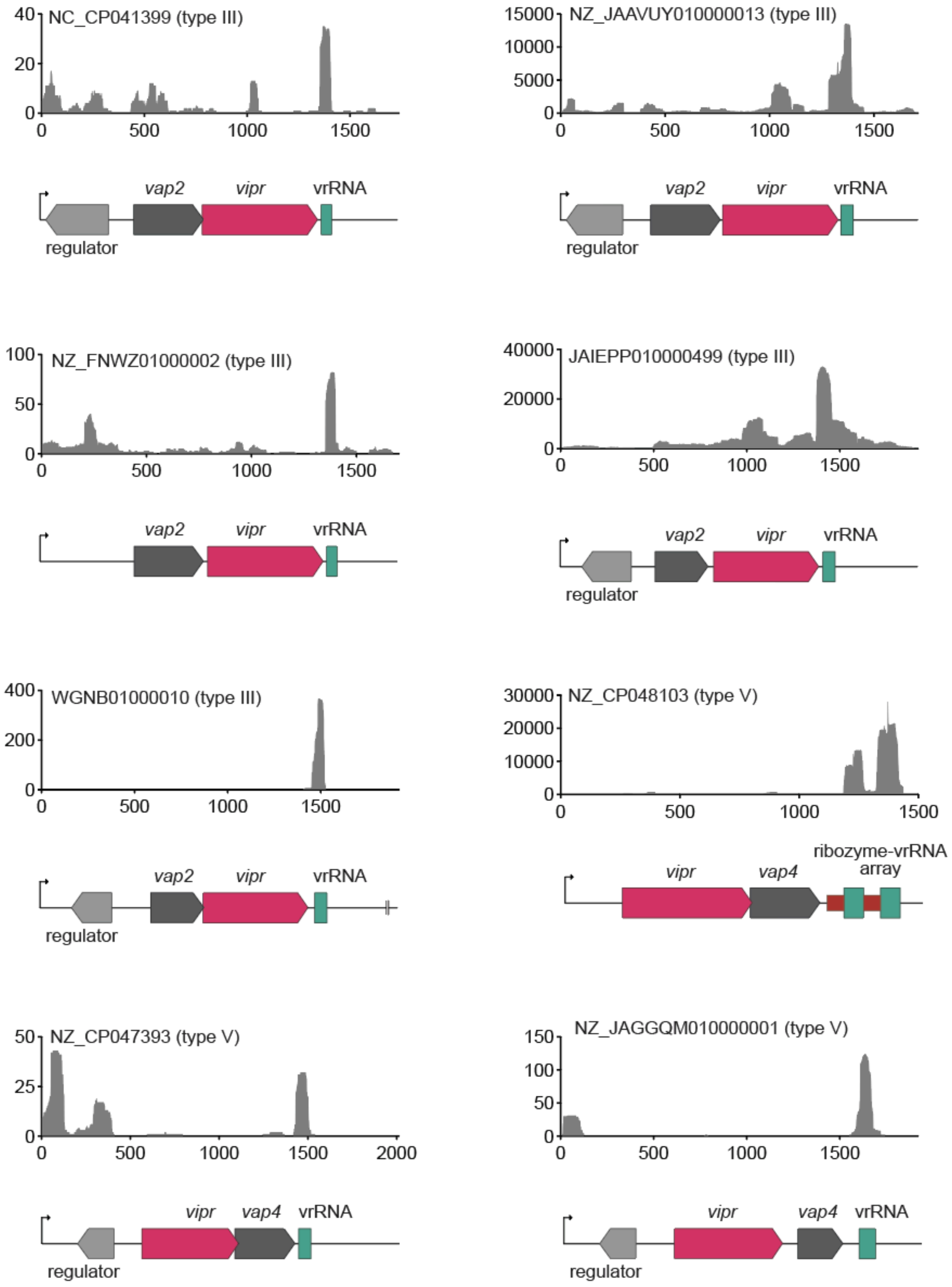

Fig. S20. sRNA-seq of type III and V VIPR systems. sRNA-seq results from *E. coli* based heterologous expression of representative type III and type V VIPR loci are shown above the corresponding locus diagrams.

bioRxiv preprint doi: https://doi.org/10.64898/2026.04.26.720920; this version posted June 25, 2026. The copyright holder for this preprint (which was not certified by peer review) is the author/funder, who has granted bioRxiv a license to display the preprint in perpetuity. It is made available under a CC-BY 4.0 International license.

Figure S21

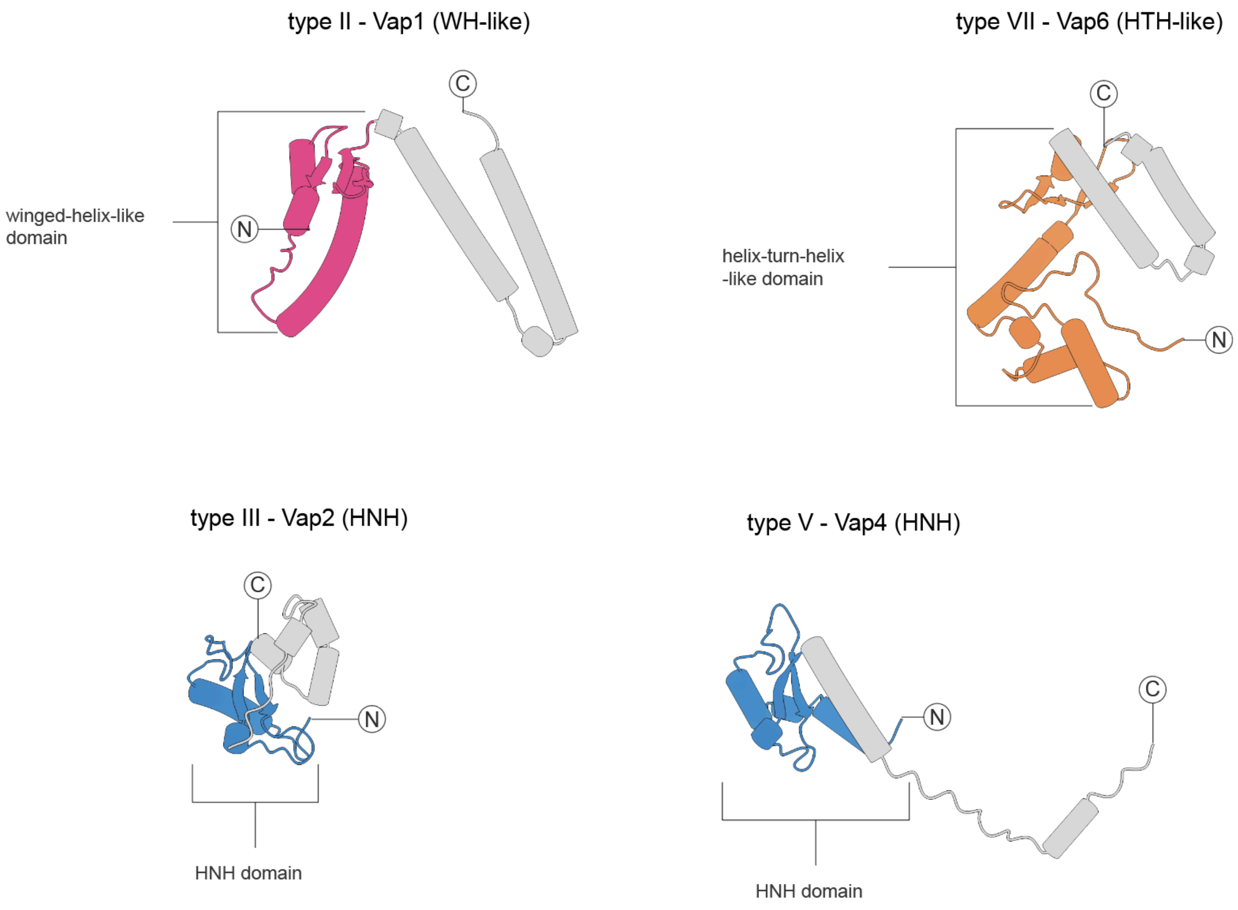

Fig. S21. Predicted DNA-binding and nuclease-associated *vap* proteins in VIPR systems. Predicted structures of representative Vap proteins from type II, III, and VII VIPR systems are shown. Putative functional domains are highlighted in color and labeled, with the remaining regions shown in gray. Examples include proteins containing winged-helix-like (WH-like), helix-turn-helix-like (HTH-like), and HNH domains.

bioRxiv preprint doi: https://doi.org/10.64898/2026.04.26.720920; this version posted June 25, 2026. The copyright holder for this preprint (which was not certified by peer review) is the author/funder, who has granted bioRxiv a license to display the preprint in perpetuity. It is made available under a CC-BY 4.0 International license.

Figure S22

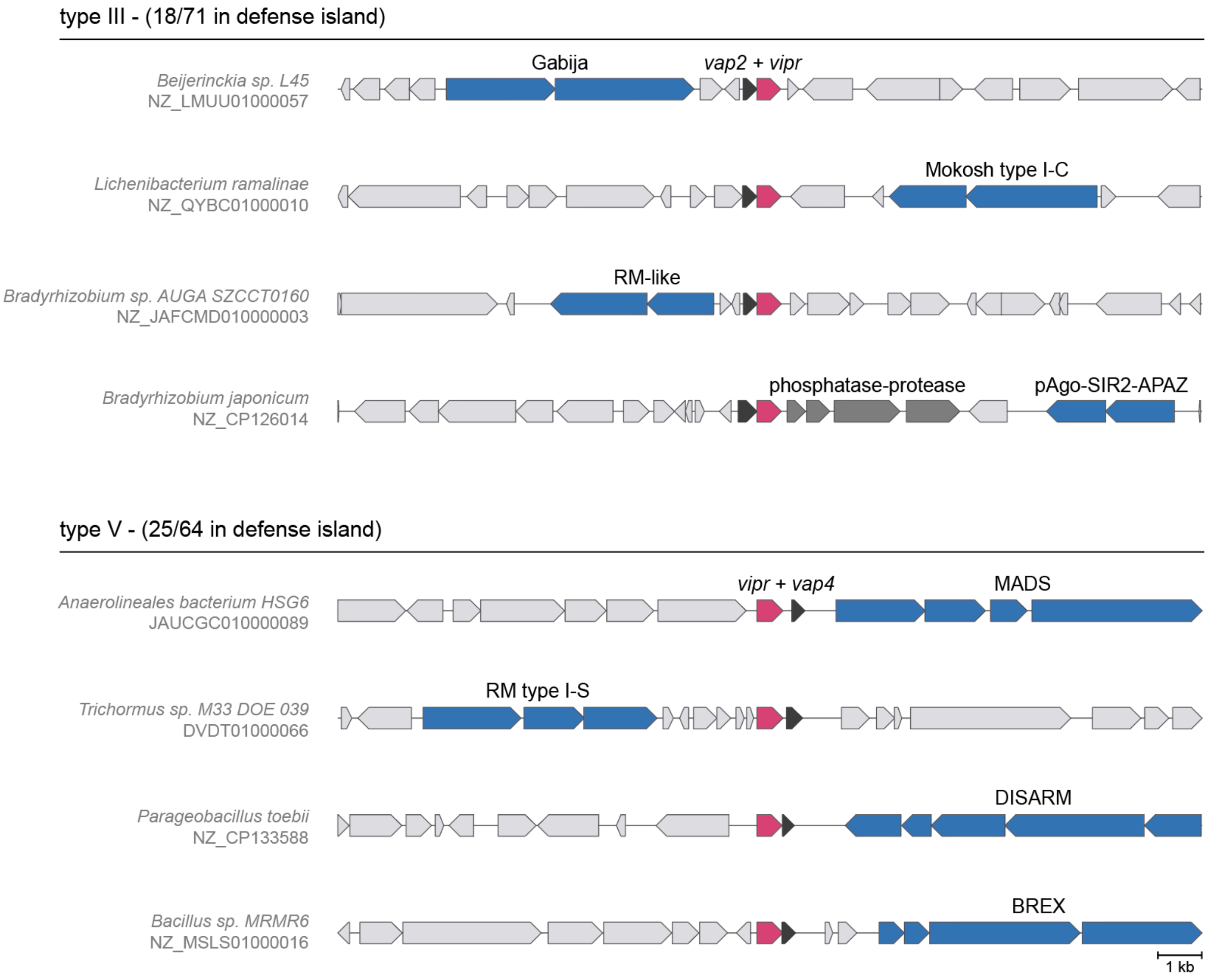

Fig. S22. Locus diagrams for representative type III and V systems. Representative genomic loci for type III and type V systems are shown. The number of loci located within defense islands is indicated for each type. Defense systems are annotated in blue.
